# Distinct profiling of antimicrobial peptide families

**DOI:** 10.1093/bioinformatics/btu738

**Published:** 2014-11-10

**Authors:** Abdullah M. Khamis, Magbubah Essack, Xin Gao, Vladimir B. Bajic

**Affiliations:** Computational Bioscience Research Center (CBRC), Computer, Electrical and Mathematical Sciences and Engineering (CEMSE) Division, King Abdullah University of Science and Technology (KAUST), Thuwal 23955-6900, Saudi Arabia

## Abstract

**Motivation:** The increased prevalence of multi-drug resistant (MDR) pathogens heightens the need to design new antimicrobial agents. Antimicrobial peptides (AMPs) exhibit broad-spectrum potent activity against MDR pathogens and kills rapidly, thus giving rise to AMPs being recognized as a potential substitute for conventional antibiotics. Designing new AMPs using current *in-silico* approaches is, however, challenging due to the absence of suitable models, large number of design parameters, testing cycles, production time and cost. To date, AMPs have merely been categorized into families according to their primary sequences, structures and functions. The ability to computationally determine the properties that discriminate AMP families from each other could help in exploring the key characteristics of these families and facilitate the *in-silico* design of synthetic AMPs.

**Results:** Here we studied 14 AMP families and sub-families. We selected a specific description of AMP amino acid sequence and identified compositional and physicochemical properties of amino acids that accurately distinguish each AMP family from all other AMPs with an average sensitivity, specificity and precision of 92.88%, 99.86% and 95.96%, respectively. Many of our identified discriminative properties have been shown to be compositional or functional characteristics of the corresponding AMP family in literature. We suggest that these properties could serve as guides for *in-silico* methods in design of novel synthetic AMPs. The methodology we developed is generic and has a potential to be applied for characterization of any protein family.

**Contact:** vladimir.bajic@kaust.edu.sa

**Supplementary information:** Supplementary data are available at *Bioinformatics* online.

## 1 Introduction

Escalating deaths due to increased exposure of living organisms to infectious diseases and the rapid evolution of multi-drug resistant (MDR) microbes spurred interest in alternative remedies that can treat the MDR microbes causing these diseases (Saxena and Gomber, 2010). Antimicrobial peptides (AMPs) have been pinpointed as one such remedy (Gordon *et al*., 2005; Hancock and Diamond, 2000; Sundararajan *et al*., 2012), owing to their broad-spectrum potent activity against Gram-positive and Gram-negative bacteria, fungi, protozoa, parasites, cancer cells and different kinds of enveloped viruses (Hancock and Scott, 2000; Zasloff, 2002; Thomas *et al*., 2010). Their effective defense action against a broad spectrum of microbes and their ability to kill rapidly have rendered them highly effective substitute for conventional antibiotics (Hancock and Lehrer, 1998; Brahmachary *et al*., 2004; Hancock and Sahl, 2006; Peters *et al.*, 2010; Thomas *et al*., 2010). Moreover, designed AMPs have been reported to kill MDR microbes (Yeaman and Yount, 2003). AMPs interact with target microbes by membrane permeation and penetration (Andreu and Rivas, 1998; Brogden, 2005; Radek and Gallo, 2007), subsequently affecting cytoplasmic membrane septum formation (additional complementary mechanisms involve disruption of cell wall, nucleic acids and proteins biosynthesis processes), thereby ultimately killing the targeted microbe (Frecer *et al.*, 2004; Peters *et al*., 2010; Chen *et al*., 2012; Fjell *et al*., 2012). Differences in the membrane structure of higher eukaryotes and microbes cause the later to be easily recognized and targeted (Thomas *et al*., 2010).

AMPs are typically short molecules of less than 100 amino acids (Jenssen *et al*., 2006; Sang and Blecha, 2008; Peters *et al.*, 2010; Pasupuleti *et al*., 2012), most of which are cationic and amphipathic (Epand and Vogel, 1999; Lehrer and Ganz, 1999). They exist in all classes of life and are evolutionarily conserved (Yeaman and Yount, 2003; Hancock and Sahl, 2006). Thus, AMPs have been classified into families and sub-families based on their primary sequences and structures (Kaiser and Diamond, 2000; Yeaman and Yount, 2003). However, there is substantial experimental evidence that even minor variations in peptide structures can lead to significant differences in AMP activities (Ganz, 2003; Thomas *et al*., 2010).

Hundreds of natural AMPs have been identified and characterized, with information in public databases, e.g. DAMPD (Sundararajan *et al*., 2012), CAMP (Thomas *et al*., 2010; Waghu *et al*., 2014), APD2 (Wang *et al*., 2009), APD (Wang and Wang, 2004) and ANTIMIC (Brahmachary *et al*., 2004). However, increased demand for AMPs fostered more interest in *in-vitro* design of AMPs (Nusslein *et al*., 2006; Marcos *et al*., 2008). The design of novel synthetic AMPs is challenging due to the wide variety of properties that characterize them (Guralp *et al.*, 2013). Thus, developing computational models that identify these essential properties and pinpoint candidate AMPs have gained interest (Fjell *et al.*, 2012; Maccari *et al*., 2013). An effective and efficient *in-silico* approach is needed to identify candidate properties that may guide the design of new AMPs (Juretic *et al*., 2011).

A number of methods have been proposed to identify and characterize AMPs using compositional characteristics of their amino acid sequences and information extracted from sequence alignment (Lata *et al*., 2007, 2010; Wang *et al.*, 2011). However, the activities and interaction mechanisms of AMPs cannot be fully characterized exclusively using their amino acid composition. Rather, their structural and physicochemical properties could help in better understanding the determinants underlying these activities (Fjell *et al*., 2012). Efforts have been made to study physicochemical properties of AMPs (Langham *et al.*, 2008; Porto *et al.*, 2010; Torrent *et al.*, 2011; Maccari *et al.*, 2013). However, a comprehensive and systematic analysis of these properties, their colocation within different AMP regions and characterization of different AMP families by them to the best of our knowledge are not yet available.

In this study, we developed a novel computational model to represent AMP sequences and a method to identify physicochemical and compositional properties of AMPs that are capable to distinguish different AMP families from each other. The methodology we developed to represent AMP families and identify their properties is generic, with a potential to be used in characterizing arbitrary protein family. The differences we identified in characterizing various AMP families by these features are so pronounced that, in a machine-learning framework, they enable for most of the AMP families 100% specificity in separation of peptides of an AMP family from other AMPs, providing sensitivity that ranges from approximately 62% to 100%. Many of our identified discriminative properties have been shown to be compositional or functional characteristics of the corresponding AMP family in literature. These results suggest that the identified AMP properties make them potential design guides for development of synthetic AMPs with characteristics of specific families.

## 2 Methods

### 2.1 Datasets

Sequences of all 753 non-redundant natural mature peptides known to belong to specific AMP families and sub-families (in our case 128 families/sub-families) were obtained from the DAMPD database (Sundararajan *et al*., 2012). All AMPs in DAMPD database were manually curated and experimentally validated to possess antimicrobial activity. The AMPs in the database were classified into families/sub-families according to their annotation provided by UniProt (2014). We examined the AMP families/sub-families that contain more than 10 peptides that we denote as ‘target AMP families’. Consequently, we studied 14 target AMP families that all together have 465 peptides. The 288 peptides that belong to the remaining 114 families/sub-families of AMPs were used as a part of the ‘negative dataset’. The negative dataset is specific for each target AMP family and contains the 288 peptides mentioned above, as well as all peptides from the remaining 13 target AMP families (excluding peptides from the target AMP family). Such negative datasets are used during the identification of properties that characterize each of the 14 target AMP families. That is, the peptides of a particular target AMP family are contrasted to all other AMP peptides in the negative dataset (Supplementary Table S1) shows the distribution of the peptides among the obtained families/sub-families. Comparison of the amino acid composition of the selected families/sub-families is provided in Supplementary Materials S7. In order to compare AMP versus non-AMPs, we also compiled as set of non-AMPs as follows:

1. We used from Uniprot all protein sequences of length 10–212 (same length distribution as the AMP sequences) whose ontology annotation does not contain any keyword related to antimicrobial activity (e.g. Antimicrobial, Antibacterial, Antifungal, Fungicide, Defensin, Antiviral, etc.). This provided 18 082 non-AMP sequences.
2. Then, to reduce redundancy among these sequences, three clustering steps using the h-cd-hit (Li and Godzik, 2006) program were made at three identity thresholds (≥90%, ≥60% and ≥30%). The purpose of this was to remove identical sequences and obtain a non-redundant dataset of non-AMP sequences. As a result, we obtained 7066 non-AMP sequences.
3. Finally, we retained those sequences that were composed only of uniquely defined canonical 20 amino acids (excluding all those with nonstandard letters B, Z, J, X, U and O). This produced 6740 non-AMP sequences.

### 2.2 Models of peptide/protein sequences

The methodology we developed to represent different peptides/proteins can be summarized as follows. Our method complements an earlier introduced method to computationally model in the same manner proteins of different lengths and families for analysis of their cellular localization (Matsuda *et al.*, 2005). This model by Matsuda *et al.* enabled that every protein sequence, irrespective of its length and family, is described in the same manner enabling their computational comparison and equal treatment. Under this model, different proteins will have different parameters of the model derived from compositional properties of different protein regions. Our extension of this model was in adding to such protein description a part with physicochemical characteristics in a way that is not dependent on the protein/peptide length (as this aspect has already been resolved by the Matsuda *et al.* model). Essentially, each of the regions defined by the Matsuda *et al.* model we associated with the restrictive physicochemical properties of that region and the GA optimization we applied selected the features from that set. Consequently, the modeling methodology we developed as explained above caters for protein/peptides of different length and their variable characteristics.

We explain this methodology as in the following. AMP sequences are divided into three regions. This is motivated by the fact that N and C terminals of the peptide sequences are enriched with properties that discriminate peptide families from each other (Hayes *et al.*, 2006; Lata *et al.*, 2010; Minervini *et al.*, 2003). Then, each peptide is encoded by a feature vector composed of two parts. The first part consists of features that represent mainly the peptide amino acid composition. The second part involves the representation of the restrictive physicochemical properties (those that show high invariance values within peptides of the same family) within the predetermined regions in the peptide sequence. These two parts of the feature vector are explained in what follows.

#### 2.2.1 Basic representation of peptide features

We represent peptides from all families using the same number of features. We followed the protein representation method proposed in Matsuda *et al*. (2005). In this representation, the peptide sequence is divided into three regions, N-terminal (N), middle region (M) and C-terminal (C). The N-terminal is further divided into four sub-regions: n1, n2, n3 and n4. The size of each region is determined depending on the sequence length *L*. That is, long sequences will have longer N and C termini, whereas short sequences will have shorter termini. For the reason that AMP peptides are of shorter length when compared with most other proteins and because peptides among AMP families differ between each other in the length of their sequences, we tested the performance of the developed model using different values of *d*_N_ (length of one of the four sub-regions within the N-terminal) and *d*_C_ (length of C-terminal) parameters for each family. We selected the *d*_N_ and *d*_C_ values (Table 1 and Supplementary Materials S2.3) that we found through experimentation to be the most suitable for our intended analysis. Then, the sequence of a peptide is represented by a 184-feature vector as follows: 140 features represent the composition of amino acids in all the regions n1, n2, n3, n4, M, C and the entire sequence (20 features in 7 regions); 20 features for the composition of twin amino acids (two successive same amino acids, e.g. AA, LL) in the M-region; 6 features for the distance frequencies of basic amino acids (R, K and H) in each of N and M regions giving in total 12 features; another 6 features for the distance frequencies of hydrophobic amino acids (I, V, L, F, M, A, G, W and P) in the M-region and the last 6 features for the distance frequencies of other amino acids (D, N, E, Q, Y, S, T and C) in the M-region. The six-values of distance frequencies are calculated as follows. The distance (*H*) between two successive amino acids in the specified class is assigned to one of six distance categories (*H* = 1, 1 < *H* ≤ 6, 6 < *H* ≤ 11, 11 < *H* ≤ 16, 16 < *H* ≤ 21, *H* > 21). Then, the number of occurrences of the distances in these categories represents the distance frequencies. For example, the distances between basic amino acids (R, K and H) in the sequence (ARMRAASKAALLMAHKNAK) are 2, 4, 7, 1 and 3. The six frequency values of these distance values are (1, 3, 1, 0, 0 and 0). The motivation behind this representation (Matsuda *et al*., 2005) is that dividing the peptide sequence into regions gives more flexibility to capture the peptide signal sequences, and such signals might be distributed across different regions of the peptide sequence.

**Table 1.** The number of features characterizing different AMP sequence regions as selected using GA-based optimization of unsupervised *k*-means clustering

AMP family/sub-family	*d*_n_	*d*_c_	NP	NF	NSF	NC	NCF	NPF
Alpha-defensin	10	10	34	299	14	14	12	2
Bacteriocin	14	10	24	225	9	12	7	2
Beta-defensin	10	10	41	261	36	14	29	7
Bombinin	16	10	31	1095	13	9	4	9
Cathelicidin	16	8	27	521	36	11	17	19
Cecropin	12	8	30	835	33	11	8	25
Cyclotide (Bracelet)	12	10	12	350	7	14	2	5
DEFL	12	8	37	252	26	14	20	6
FSAP (Brevinin sub-family)	14	10	143	1945	118	12	18	100
FSAP (Caerin sub-family)	14	10	11	1946	28	14	1	27
FSAP (Dermaseptin)	16	10	30	327	25	15	16	9
Invertebrate def. (Type 1)	10	10	21	402	14	14	8	6
Invertebrate def. (Type 2)	16	8	13	510	9	15	4	5
Type A lantibiotic	16	10	11	194	26	14	26	0
**Total**				9162	394		172	222

*Notes:* Annotations of columns are as follows: N-terminal length (*d*_n_), C-terminal length (*d*_c_), number of peptides (NP), original number of features (NF), number of selected features (NSF), number of clusters (NC), number of compositional features (NCF) and number of physicochemical features (NPF).

#### 2.2.2 Adding family-specific features

AMP families differ among each other in the set of restrictive properties found in different regions of their peptide sequences. Different regions may have different restrictive properties. To select these restrictive properties, we used 544 physicochemical properties of amino acids available in the AAIndex database version 9.1 (Kawashima and Kanehisa, 2000). This set was further reduced to 294 properties by selecting a single (randomly selected) property from those subsets of properties that have mutual Pearson correlation coefficient of 0.9 or higher. This has eliminated the use of multiple highly correlated properties. To facilitate feature extraction, we first aligned the peptide sequences of a family using progressive multiple sequence alignment algorithm (Thompson *et al*., 1994). Then, the restrictive physicochemical properties among the aligned peptides were determined by excluding one peptide from the family at a time. Then, we examined whether the property value of all amino acids in each of the n1, n2, n3, n4, M and C regions of the excluded peptide is within the min/max values for the same property determined from the other peptides of the same family in that region. This test was repeated for all peptides (leave one out test). Subsequently, physicochemical properties in each region that are restricted for at least 90% of family peptides were selected to represent that region. Eventually, the median values of these properties for individual amino acids in the region entered the feature vector. This method to identify the restrictive physicochemical properties is explained as in the algorithm available in Supplementary Materials S1.1. This algorithm seeks to identify ‘restrictive’ physicochemical properties in each region of AMP peptides from a specific family.

### 2.3 Data preparation

#### 2.3.1 Normalization

To remove the bias that arises from different ranges of peptide feature values, we normalized each feature as follows:

(1) $${x^{\prime }}_{i}=\frac{x_{i}-{µ}_{i}}{\sigma_{i}},$$

where *x_i_* is the original feature value, and *x′_i_* is the value after normalization; *μ_i_* is the mean of the feature values *x_i_* across all AMP peptides and *σ_i_* is the standard deviation.

#### 2.3.2 Data filtering

We removed from the feature vectors those that have constant values among peptides of all families.

#### 2.3.3 Target and non-target classes

We performed unsupervised *k*-means clustering to identify discriminant features of a particular AMP family. Here, the peptides of the target AMP family under study represent the target class, whereas peptides from all other families (i.e. those from the remaining 13 target AMP families and the 291 peptides of the other 114 AMP families) represent the non-target class. This process is repeated independently for each of the 14 target AMP families.

### 2.4 Selection of AMP family-specific features

To identify compositional and physicochemical properties that distinguish peptides of a particular target AMP family from all other AMPs we performed global optimization to select a set of features based on a genetic algorithm (GA) aimed at minimizing the following fitness function:

(2) F=1−Fmeasure+Regularization,

where

(3) $$F_{measure}=\frac{2*(\text{precision}*\text{recall})}{\left(\text{precision+recall}\right)}=\frac{2*\text{TP}}{2*\text{TP}+\text{FN}+\text{FP}}$$

(4) $$\text{Regularization}=\frac{\text{Number of Selected Feasures}}{\text{Total Number of Feasures}}.$$

We ran the GA with a population size of 1000 to find global optimum within 1000 generations. The crossover rate was set to 0.8 and mutation rate to 0.01. In each generation, the fitness value of each individual was evaluated. The *F*-measure was calculated by performing unsupervised clustering using the *k*-means clustering algorithm with Euclidean distance. We used the known class label of each peptide to evaluate the clustering performance by calculating the true positives (TPs), true negatives (TNs), false positives (FPs) and false negatives (FNs). These quantities are used later to calculate the *F*-measure. To remove the bias caused by initial random selection of cluster centroids, we initialized the centroids of clusters using the means of points in the positive and negative classes. We performed clustering using different number of clusters ranging from 2 to 15 and selected the number of clusters that gave the highest *F*-measure value. For optimum clustering results, all peptides of the target AMP family under study are grouped in a single cluster that represents the target class, whereas peptides from all other AMP families may reside in one or more non-target class clusters. The regularization constraint is added to encourage the optimization algorithm to select the minimum number of features that yield the highest *F*-measure value. We emphasize that we do not aim at building a predictive model for the families of AMP peptides, but use this procedure to identify features that allow for the accurate clustering of peptides into AMP families.

### 2.5 Clustering AMPs into antimicrobial families

We examined the capability of the selected properties for each target AMP family to group the family peptides together and discriminate them from all other AMPs. For the purpose of testing a specific family *X*, we represented peptides of all families using the set of features selected for family *X* and successively performed clustering using *k*-means clustering with Euclidean distance. In the optimal case, all peptides of family *X* should be grouped in one cluster while peptides of other families can be distributed in one or more clusters. Based on this testing criterion, we measured the performance of clustering for the set of identified properties.

### 2.6 Model evaluation

After clustering AMPs, the cluster containing the maximum number of peptides of the target class was selected as the cluster of the target class. The remaining non-target clusters may contain AMPs from either non-target or target class. Therefore, the quality of clustering was evaluated using accuracy, sensitivity, specificity, precision, Jaccard index and *F*-measure. We used two more evaluation measures defined in Tan *et al.* (2006), the entropy and purity. All these measures are defined as in Supplementary Materials S1.2. All modules of this computational model were developed using MATLAB (R2012b).

## 3 Results

### 3.1 Feature selection using global optimization of unsupervised k-means clustering

Peptides of a particular AMP family are described by two sets of features (see Section 2). The first set consists of 184 features that represent mainly the amino acid composition in different regions of the peptide sequences. The second set is composed of restrictive physicochemical properties in different regions of the peptide sequences. The entire set of features, used to represent peptides of a particular family, contributes in different ways to distinguish one AMP family from other AMP families. Consequently, we performed feature selection using a GA for global optimization of unsupervised clustering. The GA is more suitable for optimization of discrete variables and outperforms other methods such as Particle Swarm Optimization (Kennedy, 2010) and differential evolution (DE) (Chakraborty, 2008) as shown in Supplementary Materials S3.1. Our analysis shows that *k*-means algorithm provided better clustering results when compared with other algorithms, e.g. Affinity Propagation (AP) (Frey and Dueck, 2007) (Supplementary Materials S3.2). Also, we compared the clustering results using different distance measures (Euclidean distance, correlation, cosine and city block). *K*-means clustering with Euclidean distance yielded better performance than other measures (Supplementary Materials S3.3) because AMPs have varying lengths of their sequences, as shown in Supplementary Figure S1, we performed feature selection for each AMP family using different lengths of N and C terminals, i.e. *d*_n_ = 10, 12, 14, 16 and *d*_c_ = 8, 10. Supplementary Materials S2.3 show the clustering performance using all combinations of terminal length parameters. Furthermore, feature selection was performed using different number of clusters (*K*) where K=2,3,… ,15. Columns 2–7 in Table 1 show the values of the parameters that produced the highest *F*-measure value.

As shown in Table 1, from the entire set of 9162 features used to represent all peptides of the 14 target AMP families, a subset of 394 features was selected to distinguish peptides of each AMP family from any other AMP.

### 3.2 Clustering AMPs using selected feature subsets

We used the subset of features by GA as characterizing each target AMP family to perform clustering and test if the peptides of that family will fall in one cluster while other AMPs would fall in other clusters. We measured the performance of the clustering using the known labels of the peptides. Table 2 shows these clustering results for each target AMP family. We notice the remarkable capability of the selected features to produce correct clustering with 100% specificity for 9 of the 14 AMP families, whereas for the remaining five families specificity values were between 99.17% and 99.86%. The obtained average values of sensitivity, specificity and precision for all 14 target AMP families were 92.88%, 99.86% and 95.96%, respectively. Supplementary Figure S2 shows sensitivity versus precision for the clustering obtained with the selected features.

**Table 2.** The performance of the *k*-means clustering of 14 target AMP families using features selected by GA

Target AMP family	Number of features	Accuracy (%)	Sensitivity (%)	Specificity (%)	Precision (%)	*F*-Measure (%)
Alpha-defensin	14	99.73	94.12	100.00	100.00	96.97
Bacteriocin	9	99.87	95.83	100.00	100.00	97.87
Beta-defensin	36	99.60	95.12	99.86	97.50	96.30
Bombinin	13	100.00	100.00	100.00	100.00	100.00
Cathelicidin	36	98.80	88.89	99.17	80.00	84.21
Cecropin	33	100.00	100.00	100.00	100.00	100.00
Cyclotide (Bracelet)	7	100.00	100.00	100.00	100.00	100.00
DEFL	26	99.47	89.19	100.00	100.00	94.29
FSAP (Brevinin)	118	95.88	79.02	99.84	99.12	87.94
FSAP (Caerin)	28	100.00	100.00	100.00	100.00	100.00
FSAP (Dermaseptin)	25	99.60	96.67	99.72	93.55	95.08
Invertebrate def. (Type 1)	14	100.00	100.00	100.00	100.00	100.00
Invertebrate def. (Type 2)	9	99.34	61.54	100.00	100.00	76.19
Type A lantibiotic	26	99.47	100.00	99.46	73.33	84.62
Average		99.41	92.88	99.86	95.96	93.82

*Note*: The values of other measures (Jaccard index, entropy and purity) are shown in the detailed table available in Supplementary Table S2.

Results in Table 2 revealed the advantages of characterizing peptide sequences using physicochemical properties. The long distance interactions between residues in the peptide sequence and similarity in function between distantly related proteins are likely captured at least partly using physicochemical properties (Du and Li, 2006; Liu *et al*., 2012). Consequently, using merely the information about amino acid composition may be insufficient to distinguish AMPs, determine their specificity toward target cells and characterize their activities (Maccari *et al*., 2013; Pushpanathan *et al.*, 2013). To test the importance of physicochemical properties in characterizing AMP peptide families, we represented AMP peptides using 184 features (part1 of the feature vector only) that correspond to information mainly about amino acid composition and show in Supplementary Table S3 the results of clustering with this representation. The obtained average values of sensitivity, specificity and precision for all 14 target AMP families were 73.08%, 94.50% and 40.65%, respectively. Sensitivity and precision are significantly weaker than when using selected restrictive physicochemical and compositional properties (Table 2).

We additionally demonstrated that clustering using both parts of the feature vector (i.e. the restrictive features along with the compositional features), but without feature subset selection, did not discern well peptides of any AMP family, as shown in Supplementary Table S4. The obtained average values of sensitivity, specificity and precision for all 14 target AMP families were 73.55%, 91.74% and 31.12%, respectively. Again, sensitivity and precision are significantly weaker than when we used selected restrictive physicochemical and compositional properties (Table 2). These findings showed the merit of feature subset selection to identify family-specific amino acid composition and physicochemical properties that characterize the peptides of the family and discriminate the AMP family from other AMPs.

**Fig. 1.**
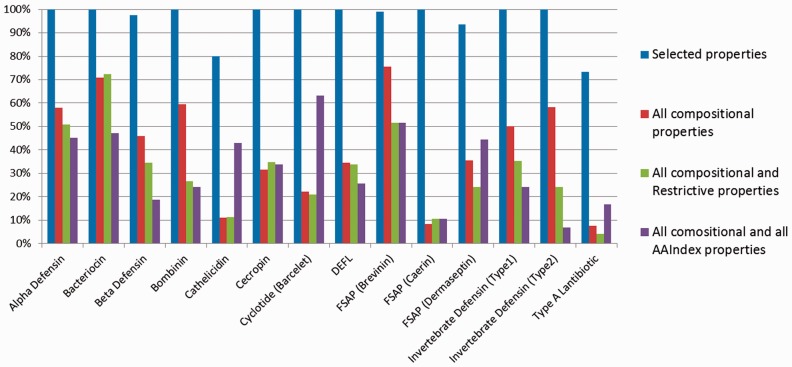
Bar plots of the precision obtained from four different representations of AMPs

A characteristic of our representation of peptide sequences is the identification of restrictive physicochemical properties in different regions of the peptide sequence. To compare this representation with the simple method of using all compositional and physicochemical properties in different regions of the peptide sequence, we used the entire set of 294 amino acids features in each of the six regions (n1, n2, n3, n4, M and C), that has resulted into a total of 184 + (294 × 6) = 1948 features without making any further feature selection. The results of clustering are shown in Supplementary Table S5. The obtained average values of sensitivity, specificity and precision for all 14 target AMP families were 63.91%, 93.54% and 32.51%, respectively. Once again, sensitivity and precision are significantly lower than when features selected by our method are used (Table 2), thereby affirming the importance of identifying the restrictive physicochemical properties in different regions of the peptide sequence and, then, using feature selection from this subset of restrictive properties. This is because the majority of properties do not contribute to distinguishing the AMP families and using them affects the values of the distance measure. Figure 1 and Supplementary Figure S3 compare the precision and the sensitivity obtained by four different methods used to represent the AMPs.

### 3.3 Testing the selected properties on non-AMPs and other AMP databases

We checked whether the features determined for AMP families would separate AMPs from non-AMPs. If this is not possible, then the non-AMPs would have similar feature profiles as some classes of AMPs. We found that this distinguishing of AMPs from non-AMPs was possible with an average accuracy, sensitivity, specificity and cluster purity of 96.72%, 67.62%, 96.85% and 99.56%, respectively, confirming that features selected for AMP families are highly specific to AMPs. One should note that our selection of features was not made with the aim to distinguish AMP families from non-AMPs. Rather, they were selected to distinguish different AMP families from other AMPs.

Furthermore, we evaluated another database CAMP (Waghu *et al.*, 2014). We considered only experimentally validated mature AMP peptide sequences with UNIPROT IDs from CAMP. We tested if the selected features based on DAMPD database entries would separate AMP families from CAMP and found that this is possible with an average accuracy, sensitivity, specificity and cluster purity of 94.03%, 76.91%, 94.58% and 97.96%, respectively. This suggests that the features selected based on DAMPD entries discriminate well between different AMP families in the CAMP database. It should be mentioned, however, that the criteria for inclusion of AMPs into CAMP and into DAMPD are not necessarily the same, so very strict comparison is not possible.

### 3.4 Selected properties that discriminate AMP families

In total, 394 properties were identified to discriminate each of the 14 target AMP families from all other AMPs (Table 1, Column 6). The entire set of the selected properties is provided in Supplementary Materials S6. Different numbers of properties were identified as being characteristic for different families. For example, seven properties could discriminate cyclotides (bracelet subfamily) from all other AMPs with an accuracy of 100%. However, more properties were needed to discriminate some AMP families from other AMPs, in particular sub-families of the same super family. For example, sub-families of the frog skin active peptide (FSAP) family such as brevinin, caerin and dermaseptin required 118, 28 and 25 properties, respectively.

The selected properties are region-specific within the peptide sequence. That is, the properties are identified from the N-terminal, M region, C-terminal or the entire peptide sequence. Some of the selected properties are related to amino acid sequence composition features, whereas some others are related to physicochemical properties. The distribution of the selected properties between these two categories of features is shown in Table 1, Columns 8 and 9. Amino acid composition properties are capable of discerning highly differential AMP family from other families, such as in the case of type A lantibiotic. However, other 13 families require a combination of amino acid composition and physicochemical properties to discern their peptides from other AMPs.

Some of the selected properties indicate the enrichment of a particular residue(s) or physicochemical property in a specific region, these properties are called ‘enriched’. However, some other properties indicate the importance of the absence or depletion of a particular residue(s) or physicochemical property in a specific region, which we call them ‘depleted’ properties. For example, among the selected properties for alpha defensins, the enrichment of arginine and the depletion of lysine in the C-terminal were identified as important properties for this family.

## 4 Discussion

In this work, we developed a novel computational model for selection of AMP characteristics that discern AMP families via physicochemical and compositional properties. The identified AMP properties make them potential design guides for development of synthetic AMPs. Also, these properties can be used as a starting point to develop a classification model to determine the category of a new candidate AMP. In the subsequent text, enrichment and depletion are considered related to one target AMP family relative to all other AMP families. Here we comment on the properties selected for some of the AMP families. Discussion of properties of other families is in Supplementary Materials S5.

*Alpha-defensins*. We found enrichment of cysteine (C) in the n3 and n4 regions, arginine (R) in the M region and arginine and tyrosine (Y) in the C region, whereas glycine (G) was found depleted in the n2 region as well as lysine (K) in the C region. Numerous studies have demonstrated that bactericidal activity is independent of highly conserved features, such as invariant disulfide array, Arg-Glu salt bridge or Gly residue at CysIII+8 I, with exception to the high arginine content relative to lysine (Lehrer, 2007; Rajabi *et al.*, 2008; Schmidt *et al.*, 2012), which complies with our finding. Moreover, Schmidt *et al*. (2012) demonstrated that the replacement of arginine with lysine decreases the activity of these peptides (Schmidt *et al.*, 2012). Also, AMPs disrupt membranes through a combination of electrostatic interactions between cationic amino acid side chains and electronegative components of the microbial cell envelope, followed by the insertion of hydrophobic patches into the nonpolar interior of the membrane bilayer (Brogden, 2005). The mouse alpha-defensin cryptdin-4 (Crp4) was demonstrated to induce bactericidal activity via this mechanism (Satchell *et al*., 2003). NMR structure of Crp4 demonstrated its cationic amino acids to be arginine, lysine and histidine (H) and its hydrophobic patches to include isoleucine (I), leucine (L), valine (V), phenylalanine (F) and tyrosine. Similar to these findings, we found for alpha-defensins the enrichment of cysteine in the N-region, which, taking into account their hydropathy index, may suggest that it is a key component of the hydrophobic patch. The enrichment of arginine and tyrosine likely adds to the electrostatic interaction that contributes to membrane disruption.

*Beta-defensins*. We found arginine, valine, phenylalanine, asparagine (N), cysteine and glycine enriched in the n1, n2, n3, n4, n4 and n4 regions, respectively. Also, for the properties extracted from the n4 sub-region, an enrichment for the parameter of charge transfer donor capacity was found (Charton and Charton, 1983). The M region exhibited enrichment, of cysteine, whereas the C region had enrichment of arginine and cysteine. It was proposed that the beta-defensin N-terminal helix with many hydrophobic residues is inserted inside the micelle, whereas the C-terminal helix with one large positive charge patch is located outside the micelle and interacts with the charged head groups of the micelle (Chandrababu *et al.*, 2009). Of the array of amino acids, we identified valine, phenylalanine and cysteine as key hydrophobic residues enriched in the N-terminal that likely facilitate the insertion of the beta-defensins N-terminal helix into the micelles, whereas for the C-terminal helix arginine is the key amino acid forming the positive charge patch. Finally, if we only consider amino acids that our study identified as enriched, we observe cysteine, glycine and arginine most enriched. Our findings are partly corroborated by the results of Midorikawa *et al*. and Chandrababu *et al*. as they demonstrated that the beta-defensins are characterized by the enrichment of cysteine. That is the existence of six conserved cysteine residues (Midorikawa *et al.*, 2003) and that the arrangement of cysteine residues in the three-dimensional space are important to the antimicrobial selectivity and salt-dependent activity by mutating all six cysteine residues of human beta-defensin-3 (HBD-3) (Chandrababu *et al.*, 2009).

*Cathelicidins.* We identified the enrichment of arginine in the n1 and n2 regions. Lysine was identified depleted in the C-region, whereas proline (P) was enriched in the C-region. Also, for the properties extracted from the n3 sub-region enrichment for the linker propensity from 1-linker dataset and normalized frequency of beta-sheet with weights was found (Levitt, 1978; George and Heringa, 2002). Our findings are supported by cathelicidins being characterized as proline-rich and having a highly conserved N-terminal preprosequences followed by variable C-terminal sequences that are biologically active effectors (Zanetti *et al.*, 1995; Chan *et al.*, 2001). Moreover, the proline was proved to sustain the antimicrobial activity of mammalian cathelicidins by resisting serine proteases cleavage of the scissile bond (Shinnar *et al*., 2003). Our findings are further supported by cathelicidins from hagfish exhibiting four arginines positioned between the cathelin domain and the antimicrobial sequences (Uzzell *et al*., 2002). The arginine tetrads of these latent zymogens are believed to be specifically processes by prohormone convertases such as furin proteases in specific cells as an activity switch (Steiner, 1998; Rockwell *et al*., 2002).

*Cecropins.* We identified enrichment of lysine and glutamic acid (E) in the n3 and n4 regions, respectively, whereas alanine was enriched in the C-region. Andreu *et al*. (1983) produced synthetic cecropin A that induces comparable antibacterial activity and is indistinguishable by chemical and physical criteria from the naturally occurring cecropin A. In partial corroboration with our findings, it has been demonstrated that cecropin analogs with an impaired N-terminal helix, such as cecropin A-(3-37) with removed lysine and tryptophan has reduced membrane disrupting abilities that correlate with their lower antibacterial activity that was rationalized in terms of reduced binding to bacteria (Andreu *et al*., 1983; Steiner *et al.*, 1988). Similarly, Fink *et al*. (1989) demonstrated via a chemically synthesized cecropin D analog (9-37) that no activity is observed without phenylalanine and glutamic acid in the N-terminal. Moreover, Lee *et al*. (1989) demonstrated that lysine, glutamic acid and arginine are conserved in cecropins and that alanine is enriched.

*Cyclotides*. We only identified a few properties related to composition, such as enrichment of glutamic acid and glycine in the n4 and C-regions, respectively. For the properties extracted from the C-region enrichment for normalized frequency of turn in alpha + beta class was found (Palau *et al*., 1982). Work by Hermann *et al*. partially supports these finding as they demonstrated that methylation of charged glutamic acid residue of cyclotide cycloviolacin O_2_ decreased its potency 48-fold. They additionally showed conserved cysteine residues and demonstrated that acetylation of the two lysine residues also reduced the potency 3-fold (Herrmann *et al.*, 2006). Koehbach *et al*. elucidated the structure of kalata B7 to determine its associated ligand–receptor interaction. They inferred an interaction with the oxytocin receptor owing to loop 3 of kalata B7 (-CYTQGC-) being homologous to the six-residue ring sequence of oxytocin. They further exhibited the crucial role of the tyrosine and glutamine residues (loop 3) by generating mutated variants (Y replaced by A, S or F; Q was replaced by A or E), all of which were inactive or did not bind to the receptor (Koehbach *et al.*, 2013). Moreover, Rosengren *et al*. (2003) demonstrated that most cyclotides have a glycine positioned before the cysteine residue to form the *f* angle required for the type II b-turn needed for cyclization.

## 5 Conclusion

The increased demand of AMPs brought attention to *in-silico* methods to design novel AMPs. In this study, we identified properties that strongly discriminate AMPs families from each other using global optimization of unsupervised clustering by GA. Our results suggest that the identified features can be used to filter out unlikely synthetic candidate AMPs during the design process of novel AMPs. The methodology developed here is generic and with a potential to characterize arbitrary protein family.

## Funding

This work was supported by KAUST Base Research Fund of VBB and KAUST Collaborative Research Funds of X.G.

*Conflict of interest*: none declared.

## Supplementary Material

Supplementary Data
