# Supplementary material for: Distinct profiling of antimicrobial peptide families
Source: Bioinformatics. 2014 Nov 10;31(6):849–56. doi: 10.1093/bioinformatics/btu738 (PMC4380027; doi:10.1093/bioinformatics/btu738)
Supplement: Supplementary Data [file supp_31_6_849__index.html]

Distinct profiling of antimicrobial peptide families — Distinct profiling of antimicrobial peptide families — Supplementary Data 

# Distinct profiling of antimicrobial peptide families

## Supplementary Data

files

**Files in this Data Supplement:**

- Supplementary Data - xlsx file
- Supplementary Data - docx file
