# Supplementary material for: Distinct profiling of antimicrobial peptide families
Source: Bioinformatics. 2014 Nov 10;31(6):849–56. doi: 10.1093/bioinformatics/btu738 (PMC4380027; doi:10.1093/bioinformatics/btu738)
Supplement: Supplementary Data [file supp_btu738_btu738_Supplementary_Materials.docx]

**Supplementary Materials**

for

**“Distinct Profiling of Antimicrobial Peptide Families”**

Abdullah M. Khamis, Magbubah Essack, Xin Gao, Vladimir B. Bajic^#^

**S1. Supplementary Methods**

**S1.1 The algorithm used to identify restrictive physicochemical properties**

The following algorithm seeks to identify “restrictive” physicochemical properties in each region of AMP peptides from a specific family.

| Let N be the set of L peptide sequences in AMP family F_i_ such that N=(N_1_, N_2_, …, N_L_)  Let T_i_ be one of the peptide regions T=(n1, n2, n3, n4, M, C)  Let P_j_ be amino acid property in P=(P_1_, P_2_, ..., P_294_)  For each region T_i_ in T  Scores=[]  For each property P_j_ in P  For each sequence N_k_ in N  Let S be all sequences of T_i_ region in N excluding N_k_ ,S=(x_i_ , x_i_ $\in$ N & x_i_ $\neq$ N_k_)  Let Mn be a vector of minimum values of P_j_ within individual positions of S  Let Mx be a vector of maximum values of P_j_ within individual positions of S  If property value P(N_ka_) of every amino acid N_ka_ in T_i_ region of N_k_ satisfy the following  P(N_ka_) >= Mn_a_ and P(N_ka_) <= Mx_a_ , then  Scores[j , k]= 1  Otherwise  Scores[j , k]= 0  END IF  END FOR  END FOR  Let R be the vector of overall scores such that $R_{j} =\left( \sum_{k=1} Scores[j,k] \right)/k$ for each property P_j_  Select as restrictive properties in T_i_ to be those which have overall score $R_{j}\geq0.9$  END FOR |
| --- |

**S1.2 Model Evaluation**

The quality of clustering was evaluated using accuracy, sensitivity, specificity, precision, Jaccard Index and F-measure defined as follows:

|  | $Accuracy=\frac{TP+TN}{TP+FN+TN+FP}$ | (1) |
| --- | --- | --- |
|  | $Sensitivity (Recall)=\frac{TP}{TP+FN}$ | (2) |
|  | $Specificity=\frac{TN}{FP+TN}$ | (3) |
|  | $Precision=\frac{TP}{TP+FP}$ | (4) |
|  | $Jaccard Index=\frac{TP}{TP+FP+FN}$ | (5) |
|  | $Fmeasure=\frac{2*TP}{2*TP+FN+FP}$ | (6) |

TP represents the number of peptides from the target class in the target class cluster; TN represents the number of peptides from the non-target class in the non-target class clusters; FP represents the number of peptides from the non-target class that belong to the target class cluster; and FN represents the number of peptides from the target class that belong to the non-target class clusters.

We used two more evaluation measures defined in ([Tan, et al., 2006](#_ENREF_15)), the entropy and purity. The entropy is calculated as follows. First, the probability that a peptide in cluster $i$ is a member of class $j$ is defined as:

|  | $p_{ij}=\frac{m_{ij}}{m_{i}}$, | (7) |
| --- | --- | --- |

where $m_{i}$ is the number of objects in cluster $i$; $m_{ij}$ is the number of objects of class $j$ in cluster $i$. Then, the entropy of each cluster $i$ is:

|  | $e_{i}=-\sum_{j=1}^{L} p_{ij} {log}_{2}p_{ij}$, | (8) |
| --- | --- | --- |

where $L$ is the number of classes (in our case we have two classes, target and non-target). The total entropy of a set of clusters is:

|  | $e=\sum_{i=1}^{K} \frac{m_{i}}{m} e_{i}$, | (9) |
| --- | --- | --- |

where $K$ is the number of clusters and $m$ is the total number of peptides. The purity is calculated as follows:

|  | $p_{i}={max}_{j} p_{ij}$ | (10) |
| --- | --- | --- |
|  | $purity=\sum_{i=1}^{K} \frac{m_{i}}{m} p_{i}$ | (11) |

**S2. Supplementary Tables**

**S2.1 Number of peptides in each of the 14 AMP families**

**Table S1.** Distribution of peptides among 128 AMP families and sub-families obtained from DAMPD database

| AMP family/sub-family | Number of peptides |
| --- | --- |
| Alpha-defensin | 34 |
| Bacteriocin | 24 |
| Beta-defensin | 41 |
| Bombinin | 31 |
| Cathelicidin | 27 |
| Cecropin | 30 |
| Cyclotide (Bracelet sub-family) | 12 |
| DEFL | 37 |
| FSAP (Brevinin sub-family) | 143 |
| FSAP (Caerin sub-family) | 11 |
| FSAP (Dermaseptin sub-family) | 30 |
| Invertebrate defensin (Type 1 sub-family) | 21 |
| Invertebrate defensin (Type 2 sub-family) | 13 |
| Type A lantibiotic | 11 |
| All Other AMP Families (114 Families/sub-families) | 288 |
| **Total** | **753** |

**S2.2 Clustering results using the selected properties and other representations**

**Table S2.** The performance of the k-means clustering of 14 target AMP families using features selected by GA.

| Target AMP family | Number of features | Accuracy | Sensitivity | Specificity | Precision | Jaccard Index | F-Measure | Entropy | Purity |
| --- | --- | --- | --- | --- | --- | --- | --- | --- | --- |
| Alpha-defensin | 14 | 99.73% | 94.12% | 100.00% | 100.00% | 94.12% | 96.97% | 0.0165 | 99.73% |
| Bacteriocin | 9 | 99.87% | 95.83% | 100.00% | 100.00% | 95.83% | 97.87% | 0.0116 | 99.87% |
| Beta-defensin | 36 | 99.60% | 95.12% | 99.86% | 97.50% | 92.86% | 96.30% | 0.0289 | 99.60% |
| Bombinin | 13 | 100.00% | 100.00% | 100.00% | 100.00% | 100.00% | 100.00% | 0 | 100.00% |
| Cathelicidin | 36 | 98.80% | 88.89% | 99.17% | 80.00% | 72.73% | 84.21% | 0.058 | 98.80% |
| Cecropin | 33 | 100.00% | 100.00% | 100.00% | 100.00% | 100.00% | 100.00% | 0 | 100.00% |
| Cyclotide (Bracelet subfamily) | 7 | 100.00% | 100.00% | 100.00% | 100.00% | 100.00% | 100.00% | 0 | 100.00% |
| DEFL | 26 | 99.47% | 89.19% | 100.00% | 100.00% | 89.19% | 94.29% | 0.0325 | 99.47% |
| FSAP (Brevinin subfamily) | 118 | 95.88% | 79.02% | 99.84% | 99.12% | 78.47% | 87.94% | 0.1478 | 95.88% |
| FSAP (Caerin subfamily) | 28 | 100.00% | 100.00% | 100.00% | 100.00% | 100.00% | 100.00% | 0 | 100.00% |
| FSAP (Dermaseptin subfamily) | 25 | 99.60% | 96.67% | 99.72% | 93.55% | 90.62% | 95.08% | 0.0254 | 99.60% |
| Invertebrate defensin (Type 1 subfamily) | 14 | 100.00% | 100.00% | 100.00% | 100.00% | 100.00% | 100.00% | 0 | 100.00% |
| Invertebrate defensin (Type 2 subfamily) | 9 | 99.34% | 61.54% | 100.00% | 100.00% | 61.54% | 76.19% | 0.0426 | 99.34% |
| Type A lantibiotic | 26 | 99.47% | 100.00% | 99.46% | 73.33% | 73.33% | 84.62% | 0.0167 | 99.47% |
| **Average** |  | 99.41% | 92.88% | 99.86% | 95.96% | 89.19% | 93.82% | 2.71% | 99.41% |

**Table S3.** The performance of the k-means clustering of 14 target AMP families using 184 features that represent information about amino acid composition.

| Target AMP family | Number of features | Accuracy | Sensitivity | Specificity | Precision | Jaccard Index | F-Measure | Entropy | Purity |
| --- | --- | --- | --- | --- | --- | --- | --- | --- | --- |
| Alpha-defensin | 184 | 96.55% | 85.29% | 97.08% | 58.00% | 52.73% | 69.05% | 0.1085 | 96.55% |
| Bacteriocin | 184 | 98.54% | 91.67% | 98.77% | 70.97% | 66.67% | 80.00% | 0.0589 | 98.54% |
| Beta-defensin | 184 | 93.89% | 68.29% | 95.37% | 45.90% | 37.84% | 54.90% | 0.1646 | 94.56% |
| Bombinin | 184 | 96.81% | 70.97% | 97.92% | 59.46% | 47.83% | 64.71% | 0.1248 | 96.81% |
| Cathelicidin | 184 | 87.12% | 37.04% | 88.98% | 11.11% | 9.35% | 17.09% | 0.1983 | 96.41% |
| Cecropin | 184 | 91.37% | 100.00% | 91.01% | 31.58% | 31.58% | 48.00% | 0.1135 | 96.02% |
| Cyclotide (Bracelet subfamily) | 184 | 94.42% | 100.00% | 94.33% | 22.22% | 22.22% | 36.36% | 0.0548 | 98.41% |
| DEFL | 184 | 92.70% | 54.05% | 94.69% | 34.48% | 26.67% | 42.11% | 0.1928 | 95.09% |
| FSAP (Brevinin subfamily) | 184 | 90.17% | 71.33% | 94.59% | 75.56% | 57.95% | 73.38% | 0.3618 | 90.17% |
| FSAP (Caerin subfamily) | 184 | 87.92% | 72.73% | 88.14% | 8.33% | 8.08% | 14.95% | 0.0809 | 98.54% |
| FSAP (Dermaseptin subfamily) | 184 | 93.23% | 86.67% | 93.50% | 35.62% | 33.77% | 50.49% | 0.1267 | 96.02% |
| Invertebrate defensin (Type 1 subfamily) | 184 | 97.21% | 85.71% | 97.54% | 50.00% | 46.15% | 63.16% | 0.075 | 97.21% |
| Invertebrate defensin (Type 2 subfamily) | 184 | 98.54% | 53.85% | 99.32% | 58.33% | 38.89% | 56.00% | 0.0656 | 98.54% |
| Type A lantibiotic | 184 | 91.10% | 45.45% | 91.78% | 7.58% | 6.94% | 12.99% | 0.0907 | 98.54% |
| **Average** |  | 93.54% | 73.08% | 94.50% | 40.65% | 34.76% | 48.80% | 12.98% | 96.53% |

**Table S4.** The performance of the k-means clustering of 14 target AMP families using the entire set of features to represent each family.

| Target AMP family | Number of features | Accuracy | Sensitivity | Specificity | Precision | Jaccard Index | F-Measure | Entropy | Purity |
| --- | --- | --- | --- | --- | --- | --- | --- | --- | --- |
| Alpha-defensin | 299 | 95.62% | 85.29% | 96.11% | 50.88% | 46.77% | 63.74% | 0.1182 | 95.62% |
| Bacteriocin | 225 | 98.54% | 87.50% | 98.90% | 72.41% | 65.62% | 79.25% | 0.0659 | 98.54% |
| Beta-defensin | 261 | 90.44% | 85.37% | 90.73% | 34.65% | 32.71% | 49.30% | 0.1752 | 94.56% |
| Bombinin | 1095 | 89.77% | 83.87% | 90.03% | 26.53% | 25.24% | 40.31% | 0.1486 | 95.88% |
| Cathelicidin | 521 | 88.31% | 33.33% | 90.36% | 11.39% | 9.28% | 16.98% | 0.1949 | 96.41% |
| Cecropin | 835 | 93.23% | 80.00% | 93.78% | 34.78% | 32.00% | 48.48% | 0.1457 | 96.02% |
| Cyclotide (Bracelet subfamily) | 350 | 94.02% | 100.00% | 93.93% | 21.05% | 21.05% | 34.78% | 0.0562 | 98.41% |
| DEFL | 252 | 92.43% | 56.76% | 94.27% | 33.87% | 26.92% | 42.42% | 0.1699 | 95.09% |
| FSAP (Brevinin subfamily) | 1945 | 81.54% | 43.36% | 90.49% | 51.67% | 30.85% | 47.15% | 0.4433 | 83.53% |
| FSAP (Caerin subfamily) | 1946 | 91.63% | 63.64% | 92.05% | 10.61% | 10.00% | 18.18% | 0.0786 | 98.54% |
| FSAP (Dermaseptin subfamily) | 327 | 89.51% | 76.67% | 90.04% | 24.21% | 22.55% | 36.80% | 0.1584 | 96.02% |
| Invertebrate defensin (Type 1 subfamily) | 402 | 95.09% | 90.48% | 95.22% | 35.19% | 33.93% | 50.67% | 0.0872 | 97.21% |
| Invertebrate defensin (Type 2 subfamily) | 510 | 96.02% | 61.54% | 96.62% | 24.24% | 21.05% | 34.78% | 0.0775 | 98.27% |
| Type A lantibiotic | 194 | 71.98% | 81.82% | 71.83% | 4.13% | 4.09% | 7.86% | 0.0944 | 98.54% |
| **Average** |  | 90.58% | 73.55% | 91.74% | 31.12% | 27.29% | 40.76% | 14.39% | 95.90% |

**Table S5.** The performance of the k-means clustering of 14 target AMP families using all the 294 physicochemical properties in each of the 6 regions (n1,n2,n3,n4,M and C).

| Target AMP family | Number of features | Accuracy | Sensitivity | Specificity | Precision | Jaccard Index | F-Measure | Entropy | Purity |
| --- | --- | --- | --- | --- | --- | --- | --- | --- | --- |
| Alpha-defensin | 1948 | 94.82% | 67.65% | 96.11% | 45.10% | 37.10% | 54.12% | 0.157 | 95.48% |
| Bacteriocin | 1948 | 96.55% | 70.83% | 97.39% | 47.22% | 39.53% | 56.67% | 0.116 | 96.81% |
| Beta-defensin | 1948 | 87.92% | 36.59% | 90.87% | 18.75% | 14.15% | 24.79% | 0.241 | 94.56% |
| Bombinin | 1948 | 88.71% | 80.65% | 89.06% | 24.04% | 22.73% | 37.04% | 0.1598 | 95.88% |
| Cathelicidin | 1948 | 96.02% | 33.33% | 98.35% | 42.86% | 23.08% | 37.50% | 0.1611 | 96.41% |
| Cecropin | 1948 | 93.49% | 66.67% | 94.61% | 33.90% | 28.99% | 44.94% | 0.1487 | 96.02% |
| Cyclotide (Bracelet subfamily) | 1948 | 99.07% | 100.00% | 99.06% | 63.16% | 63.16% | 77.42% | 0.024 | 99.07% |
| DEFL | 1948 | 90.04% | 54.05% | 91.90% | 25.64% | 21.05% | 34.78% | 0.1996 | 95.09% |
| FSAP (Brevinin subfamily) | 1948 | 81.54% | 43.36% | 90.49% | 51.67% | 30.85% | 47.15% | 0.4429 | 83.53% |
| FSAP (Caerin subfamily) | 1948 | 91.63% | 63.64% | 92.05% | 10.61% | 10.00% | 18.18% | 0.0786 | 98.54% |
| FSAP (Dermaseptin subfamily) | 1948 | 95.22% | 80.00% | 95.85% | 44.44% | 40.00% | 57.14% | 0.1234 | 96.02% |
| Invertebrate defensin (Type 1 subfamily) | 1948 | 91.24% | 100.00% | 90.98% | 24.14% | 24.14% | 38.89% | 0.0921 | 97.21% |
| Invertebrate defensin (Type 2 subfamily) | 1948 | 85.13% | 61.54% | 85.54% | 6.96% | 6.67% | 12.50% | 0.0925 | 98.27% |
| Type A lantibiotic | 1948 | 96.41% | 36.36% | 97.30% | 16.67% | 12.90% | 22.86% | 0.0876 | 98.54% |
| **Average** |  | 91.99% | 63.91% | 93.54% | 32.51% | 26.74% | 40.28% | 15.17% | 95.82% |

**S2.3 Clustering performance using different values of terminal length parameters**

The following tables show the clustering performance of selected properties using different values of terminal length parameters, dn and dc. Each row in a table refers to an AMP family of the 14 target AMP families in the same order as shown in the tables in the main text and tables in section 2.1 above.

dn = 10, dc=8

| Number of peptides | Original number of features | number of selected features | Number of clusters | Accuracy | Sensitivity | Specificity | Precision | Jaccard Index | F-Measure | Entropy | Purity |
| --- | --- | --- | --- | --- | --- | --- | --- | --- | --- | --- | --- |
| **34** | 322 | 19 | 11 | 99.60% | 97.06% | 99.72% | 94.29% | 91.67% | 95.65% | 0.0239 | 99.60% |
| **24** | 216 | 15 | 9 | 99.73% | 91.67% | 100.00% | 100.00% | 91.67% | 95.65% | 0.0204 | 99.73% |
| **41** | 258 | 30 | 14 | 99.34% | 90.24% | 99.86% | 97.37% | 88.10% | 93.67% | 0.0436 | 99.34% |
| **31** | 1101 | 16 | 4 | 100.00% | 100.00% | 100.00% | 100.00% | 100.00% | 100.00% | 0 | 100.00% |
| **27** | 791 | 71 | 6 | 97.61% | 66.67% | 98.76% | 66.67% | 50.00% | 66.67% | 0.1216 | 97.61% |
| **30** | 712 | 21 | 12 | 98.94% | 100.00% | 98.89% | 78.95% | 78.95% | 88.24% | 0.0375 | 98.94% |
| **12** | 346 | 11 | 10 | 100.00% | 100.00% | 100.00% | 100.00% | 100.00% | 100.00% | 0 | 100.00% |
| **37** | 253 | 34 | 15 | 99.20% | 86.49% | 99.86% | 96.97% | 84.21% | 91.43% | 0.052 | 99.20% |
| **143** | 1941 | 346 | 11 | 88.71% | 76.22% | 91.64% | 68.12% | 56.19% | 71.95% | 0.3398 | 89.11% |
| **11** | 1942 | 54 | 15 | 99.73% | 90.91% | 99.87% | 90.91% | 83.33% | 90.91% | 0.0175 | 99.73% |
| **30** | 294 | 21 | 10 | 99.47% | 90.00% | 99.86% | 96.43% | 87.10% | 93.10% | 0.0397 | 99.47% |
| **21** | 299 | 20 | 9 | 99.87% | 95.24% | 100.00% | 100.00% | 95.24% | 97.56% | 0.0095 | 99.87% |
| **13** | 1078 | 36 | 15 | 99.20% | 69.23% | 99.73% | 81.82% | 60.00% | 75.00% | 0.0408 | 99.20% |
| **11** | 424 | 32 | 14 | 99.34% | 72.73% | 99.73% | 80.00% | 61.54% | 76.19% | 0.0372 | 99.34% |

dn = 10, dc=10

| Number of peptides | Original number of features | number of selected features | Number of clusters | Accuracy | Sensitivity | Specificity | Precision | Jaccard Index | F-Measure | Entropy | Purity |
| --- | --- | --- | --- | --- | --- | --- | --- | --- | --- | --- | --- |
| **34** | 299 | 14 | 14 | 99.73% | 94.12% | 100.00% | 100.00% | 94.12% | 96.97% | 0.0165 | 99.73% |
| **24** | 228 | 19 | 13 | 99.87% | 95.83% | 100.00% | 100.00% | 95.83% | 97.87% | 0.0094 | 99.87% |
| **41** | 261 | 36 | 14 | 99.60% | 95.12% | 99.86% | 97.50% | 92.86% | 96.30% | 0.0289 | 99.60% |
| **31** | 1105 | 24 | 15 | 99.07% | 77.42% | 100.00% | 100.00% | 77.42% | 87.27% | 0.0592 | 99.07% |
| **27** | 794 | 43 | 12 | 97.34% | 44.44% | 99.31% | 70.59% | 37.50% | 54.55% | 0.1416 | 97.34% |
| **30** | 725 | 31 | 12 | 99.47% | 86.67% | 100.00% | 100.00% | 86.67% | 92.86% | 0.027 | 99.47% |
| **12** | 350 | 10 | 13 | 100.00% | 100.00% | 100.00% | 100.00% | 100.00% | 100.00% | 0 | 100.00% |
| **37** | 253 | 36 | 13 | 98.94% | 91.89% | 99.30% | 87.18% | 80.95% | 89.47% | 0.0595 | 98.94% |
| **143** | 1945 | 126 | 11 | 95.48% | 77.62% | 99.67% | 98.23% | 76.55% | 86.72% | 0.1732 | 95.48% |
| **11** | 1946 | 235 | 15 | 99.34% | 81.82% | 99.60% | 75.00% | 64.29% | 78.26% | 0.0295 | 99.34% |
| **30** | 327 | 27 | 11 | 99.60% | 96.67% | 99.72% | 93.55% | 90.62% | 95.08% | 0.0253 | 99.60% |
| **21** | 402 | 14 | 14 | 100.00% | 100.00% | 100.00% | 100.00% | 100.00% | 100.00% | 0 | 100.00% |
| **13** | 1088 | 35 | 11 | 99.07% | 69.23% | 99.59% | 75.00% | 56.25% | 72.00% | 0.0498 | 99.07% |
| **11** | 194 | 17 | 15 | 98.54% | 54.55% | 99.19% | 50.00% | 35.29% | 52.17% | 0.0595 | 98.54% |

dn = 12, dc=8

| Number of peptides | Original number of features | number of selected features | Number of clusters | Accuracy | Sensitivity | Specificity | Precision | Jaccard Index | F-Measure | Entropy | Purity |
| --- | --- | --- | --- | --- | --- | --- | --- | --- | --- | --- | --- |
| **34** | 322 | 7 | 12 | 99.47% | 88.24% | 100.00% | 100.00% | 88.24% | 93.75% | 0.0319 | 99.47% |
| **24** | 227 | 11 | 13 | 99.73% | 91.67% | 100.00% | 100.00% | 91.67% | 95.65% | 0.0195 | 99.73% |
| **41** | 271 | 30 | 14 | 99.47% | 90.24% | 100.00% | 100.00% | 90.24% | 94.87% | 0.0367 | 99.47% |
| **31** | 1092 | 16 | 13 | 100.00% | 100.00% | 100.00% | 100.00% | 100.00% | 100.00% | 0 | 100.00% |
| **27** | 789 | 81 | 15 | 97.74% | 62.96% | 99.04% | 70.83% | 50.00% | 66.67% | 0.1042 | 97.74% |
| **30** | 835 | 33 | 11 | 100.00% | 100.00% | 100.00% | 100.00% | 100.00% | 100.00% | 0 | 100.00% |
| **12** | 347 | 9 | 15 | 100.00% | 100.00% | 100.00% | 100.00% | 100.00% | 100.00% | 0 | 100.00% |
| **37** | 252 | 26 | 14 | 99.47% | 89.19% | 100.00% | 100.00% | 89.19% | 94.29% | 0.0325 | 99.47% |
| **143** | 1929 | 138 | 11 | 95.48% | 80.42% | 99.02% | 95.04% | 77.18% | 87.12% | 0.1738 | 95.88% |
| **11** | 1943 | 28 | 8 | 99.73% | 81.82% | 100.00% | 100.00% | 81.82% | 90.00% | 0.0188 | 99.73% |
| **30** | 295 | 19 | 12 | 99.47% | 90.00% | 99.86% | 96.43% | 87.10% | 93.10% | 0.0344 | 99.47% |
| **21** | 208 | 14 | 12 | 99.87% | 95.24% | 100.00% | 100.00% | 95.24% | 97.56% | 0.0082 | 99.87% |
| **13** | 795 | 15 | 12 | 99.34% | 61.54% | 100.00% | 100.00% | 61.54% | 76.19% | 0.0449 | 99.34% |
| **11** | 192 | 35 | 15 | 98.80% | 81.82% | 99.06% | 56.25% | 50.00% | 66.67% | 0.0415 | 98.80% |

dn = 12, dc=10

| Number of peptides | Original number of features | number of selected features | Number of clusters | Accuracy | Sensitivity | Specificity | Precision | Jaccard Index | F-Measure | Entropy | Purity |
| --- | --- | --- | --- | --- | --- | --- | --- | --- | --- | --- | --- |
| **34** | 299 | 18 | 8 | 99.47% | 88.24% | 100.00% | 100.00% | 88.24% | 93.75% | 0.0411 | 99.47% |
| **24** | 240 | 12 | 15 | 99.73% | 91.67% | 100.00% | 100.00% | 91.67% | 95.65% | 0.0193 | 99.73% |
| **41** | 275 | 30 | 14 | 99.34% | 87.80% | 100.00% | 100.00% | 87.80% | 93.51% | 0.0362 | 99.34% |
| **31** | 1095 | 24 | 13 | 100.00% | 100.00% | 100.00% | 100.00% | 100.00% | 100.00% | 0 | 100.00% |
| **27** | 791 | 33 | 14 | 98.01% | 48.15% | 99.86% | 92.86% | 46.43% | 63.41% | 0.1106 | 98.01% |
| **30** | 781 | 32 | 14 | 99.73% | 93.33% | 100.00% | 100.00% | 93.33% | 96.55% | 0.017 | 99.73% |
| **12** | 350 | 7 | 14 | 100.00% | 100.00% | 100.00% | 100.00% | 100.00% | 100.00% | 0 | 100.00% |
| **37** | 252 | 27 | 13 | 99.20% | 83.78% | 100.00% | 100.00% | 83.78% | 91.18% | 0.0532 | 99.20% |
| **143** | 1945 | 165 | 13 | 95.62% | 78.32% | 99.67% | 98.25% | 77.24% | 87.16% | 0.1549 | 96.15% |
| **11** | 1946 | 169 | 15 | 99.47% | 72.73% | 99.87% | 88.89% | 66.67% | 80.00% | 0.0293 | 99.47% |
| **30** | 327 | 47 | 15 | 99.47% | 96.67% | 99.59% | 90.62% | 87.88% | 93.55% | 0.0293 | 99.47% |
| **21** | 208 | 19 | 14 | 100.00% | 100.00% | 100.00% | 100.00% | 100.00% | 100.00% | 0 | 100.00% |
| **13** | 806 | 25 | 15 | 99.20% | 69.23% | 99.73% | 81.82% | 60.00% | 75.00% | 0.0427 | 99.20% |
| **11** | 194 | 30 | 14 | 99.20% | 100.00% | 99.19% | 64.71% | 64.71% | 78.57% | 0.0211 | 99.20% |

dn = 14, dc=8

| Number of peptides | Original number of features | number of selected features | Number of clusters | Accuracy | Sensitivity | Specificity | Precision | Jaccard Index | F-Measure | Entropy | Purity |
| --- | --- | --- | --- | --- | --- | --- | --- | --- | --- | --- | --- |
| **34** | 321 | 12 | 15 | 99.47% | 88.24% | 100.00% | 100.00% | 88.24% | 93.75% | 0.0352 | 99.47% |
| **24** | 210 | 13 | 10 | 99.73% | 91.67% | 100.00% | 100.00% | 91.67% | 95.65% | 0.0227 | 99.73% |
| **41** | 348 | 53 | 15 | 99.20% | 85.37% | 100.00% | 100.00% | 85.37% | 92.11% | 0.046 | 99.20% |
| **31** | 1091 | 37 | 15 | 100.00% | 100.00% | 100.00% | 100.00% | 100.00% | 100.00% | 0 | 100.00% |
| **27** | 790 | 68 | 14 | 98.41% | 77.78% | 99.17% | 77.78% | 63.64% | 77.78% | 0.0784 | 98.41% |
| **30** | 894 | 53 | 13 | 99.73% | 96.67% | 99.86% | 96.67% | 93.55% | 96.67% | 0.018 | 99.73% |
| **12** | 346 | 12 | 15 | 100.00% | 100.00% | 100.00% | 100.00% | 100.00% | 100.00% | 0 | 100.00% |
| **37** | 246 | 30 | 9 | 98.94% | 81.08% | 99.86% | 96.77% | 78.95% | 88.24% | 0.0706 | 98.94% |
| **143** | 1941 | 179 | 14 | 91.24% | 60.84% | 98.36% | 89.69% | 56.86% | 72.50% | 0.221 | 93.63% |
| **11** | 1942 | 44 | 14 | 99.87% | 90.91% | 100.00% | 100.00% | 90.91% | 95.24% | 0.0098 | 99.87% |
| **30** | 294 | 26 | 12 | 99.60% | 96.67% | 99.72% | 93.55% | 90.62% | 95.08% | 0.026 | 99.60% |
| **21** | 208 | 15 | 12 | 99.87% | 95.24% | 100.00% | 100.00% | 95.24% | 97.56% | 0.0083 | 99.87% |
| **13** | 796 | 37 | 14 | 99.34% | 69.23% | 99.86% | 90.00% | 64.29% | 78.26% | 0.0385 | 99.34% |
| **11** | 192 | 44 | 15 | 98.67% | 100.00% | 98.65% | 52.38% | 52.38% | 68.75% | 0.0278 | 98.67% |

dn = 14, dc=10

| Number of peptides | Original number of features | number of selected features | Number of clusters | Accuracy | Sensitivity | Specificity | Precision | Jaccard Index | F-Measure | Entropy | Purity |
| --- | --- | --- | --- | --- | --- | --- | --- | --- | --- | --- | --- |
| **34** | 299 | 13 | 13 | 99.47% | 88.24% | 100.00% | 100.00% | 88.24% | 93.75% | 0.0376 | 99.47% |
| **24** | 225 | 9 | 12 | 99.87% | 95.83% | 100.00% | 100.00% | 95.83% | 97.87% | 0.0116 | 99.87% |
| **41** | 354 | 58 | 14 | 98.27% | 78.05% | 99.44% | 88.89% | 71.11% | 83.12% | 0.1024 | 98.27% |
| **31** | 1095 | 17 | 10 | 100.00% | 100.00% | 100.00% | 100.00% | 100.00% | 100.00% | 0 | 100.00% |
| **27** | 793 | 47 | 13 | 97.74% | 48.15% | 99.59% | 81.25% | 43.33% | 60.47% | 0.1018 | 97.74% |
| **30** | 290 | 13 | 8 | 99.47% | 86.67% | 100.00% | 100.00% | 86.67% | 92.86% | 0.0302 | 99.47% |
| **12** | 350 | 10 | 14 | 100.00% | 100.00% | 100.00% | 100.00% | 100.00% | 100.00% | 0 | 100.00% |
| **37** | 246 | 25 | 13 | 99.34% | 86.49% | 100.00% | 100.00% | 86.49% | 92.75% | 0.0424 | 99.34% |
| **143** | 1945 | 118 | 12 | 95.88% | 79.02% | 99.84% | 99.12% | 78.47% | 87.94% | 0.1478 | 95.88% |
| **11** | 1946 | 28 | 14 | 100.00% | 100.00% | 100.00% | 100.00% | 100.00% | 100.00% | 0 | 100.00% |
| **30** | 327 | 29 | 15 | 99.60% | 96.67% | 99.72% | 93.55% | 90.62% | 95.08% | 0.025 | 99.60% |
| **21** | 208 | 19 | 15 | 100.00% | 100.00% | 100.00% | 100.00% | 100.00% | 100.00% | 0 | 100.00% |
| **13** | 807 | 27 | 15 | 99.07% | 69.23% | 99.59% | 75.00% | 56.25% | 72.00% | 0.0475 | 99.07% |
| **11** | 194 | 33 | 15 | 99.34% | 100.00% | 99.33% | 68.75% | 68.75% | 81.48% | 0.019 | 99.34% |

dn = 16, dc=8

| Number of peptides | Original number of features | number of selected features | Number of clusters | Accuracy | Sensitivity | Specificity | Precision | Jaccard Index | F-Measure | Entropy | Purity |
| --- | --- | --- | --- | --- | --- | --- | --- | --- | --- | --- | --- |
| **34** | 489 | 11 | 9 | 99.47% | 88.24% | 100.00% | 100.00% | 88.24% | 93.75% | 0.0403 | 99.47% |
| **24** | 244 | 17 | 13 | 99.60% | 87.50% | 100.00% | 100.00% | 87.50% | 93.33% | 0.0306 | 99.60% |
| **41** | 377 | 45 | 15 | 98.67% | 80.49% | 99.72% | 94.29% | 76.74% | 86.84% | 0.0808 | 98.67% |
| **31** | 1091 | 22 | 14 | 100.00% | 100.00% | 100.00% | 100.00% | 100.00% | 100.00% | 0 | 100.00% |
| **27** | 521 | 36 | 11 | 98.80% | 88.89% | 99.17% | 80.00% | 72.73% | 84.21% | 0.058 | 98.80% |
| **30** | 290 | 20 | 12 | 99.34% | 83.33% | 100.00% | 100.00% | 83.33% | 90.91% | 0.0417 | 99.34% |
| **12** | 346 | 11 | 10 | 100.00% | 100.00% | 100.00% | 100.00% | 100.00% | 100.00% | 0 | 100.00% |
| **37** | 234 | 27 | 15 | 99.20% | 83.78% | 100.00% | 100.00% | 83.78% | 91.18% | 0.0491 | 99.20% |
| **143** | 1941 | 222 | 14 | 89.64% | 58.04% | 97.05% | 82.18% | 51.55% | 68.03% | 0.2679 | 91.37% |
| **11** | 1942 | 29 | 15 | 100.00% | 100.00% | 100.00% | 100.00% | 100.00% | 100.00% | 0 | 100.00% |
| **30** | 294 | 31 | 10 | 99.47% | 96.67% | 99.59% | 90.62% | 87.88% | 93.55% | 0.0297 | 99.47% |
| **21** | 208 | 18 | 12 | 99.87% | 95.24% | 100.00% | 100.00% | 95.24% | 97.56% | 0.0094 | 99.87% |
| **13** | 510 | 9 | 15 | 99.34% | 61.54% | 100.00% | 100.00% | 61.54% | 76.19% | 0.0426 | 99.34% |
| **11** | 192 | 38 | 14 | 98.94% | 100.00% | 98.92% | 57.89% | 57.89% | 73.33% | 0.0248 | 98.94% |

dn = 16, dc=10

| Number of peptides | Original number of features | number of selected features | Number of clusters | Accuracy | Sensitivity | Specificity | Precision | Jaccard Index | F-Measure | Entropy | Purity |
| --- | --- | --- | --- | --- | --- | --- | --- | --- | --- | --- | --- |
| **34** | 482 | 24 | 14 | 99.60% | 91.18% | 100.00% | 100.00% | 91.18% | 95.38% | 0.0233 | 99.60% |
| **24** | 313 | 12 | 13 | 99.60% | 87.50% | 100.00% | 100.00% | 87.50% | 93.33% | 0.0246 | 99.60% |
| **41** | 383 | 49 | 14 | 98.41% | 75.61% | 99.72% | 93.94% | 72.09% | 83.78% | 0.099 | 98.41% |
| **31** | 1095 | 13 | 9 | 100.00% | 100.00% | 100.00% | 100.00% | 100.00% | 100.00% | 0 | 100.00% |
| **27** | 524 | 27 | 9 | 97.34% | 48.15% | 99.17% | 68.42% | 39.39% | 56.52% | 0.1284 | 97.34% |
| **30** | 290 | 13 | 10 | 99.47% | 86.67% | 100.00% | 100.00% | 86.67% | 92.86% | 0.0325 | 99.47% |
| **12** | 350 | 10 | 11 | 100.00% | 100.00% | 100.00% | 100.00% | 100.00% | 100.00% | 0 | 100.00% |
| **37** | 269 | 27 | 15 | 98.80% | 78.38% | 99.86% | 96.67% | 76.32% | 86.57% | 0.0744 | 98.80% |
| **143** | 1945 | 125 | 12 | 94.16% | 77.62% | 98.03% | 90.24% | 71.61% | 83.46% | 0.2072 | 94.82% |
| **11** | 1946 | 101 | 14 | 99.87% | 100.00% | 99.87% | 91.67% | 91.67% | 95.65% | 0.0066 | 99.87% |
| **30** | 327 | 25 | 15 | 99.60% | 96.67% | 99.72% | 93.55% | 90.62% | 95.08% | 0.0254 | 99.60% |
| **21** | 208 | 18 | 11 | 99.87% | 95.24% | 100.00% | 100.00% | 95.24% | 97.56% | 0.0102 | 99.87% |
| **13** | 541 | 10 | 11 | 99.07% | 53.85% | 99.86% | 87.50% | 50.00% | 66.67% | 0.049 | 99.07% |
| **11** | 194 | 26 | 14 | 99.47% | 100.00% | 99.46% | 73.33% | 73.33% | 84.62% | 0.0167 | 99.47% |

**S3. Supplementary Results**

**S3.1 Comparison between Genetic Algorithm (GA), Particle Swarm Optimization (PSO) and Differential Evolution (DE) optimization algorithms**

We compared the clustering performance using the features selected by three different global optimization algorithms, Genetic Algorithm (GA), Particle Swarm Optimization (PSO) and Differential Evolution (DE). We found the following:

1. The majority of the features selected by GA were among features selected by PSO and DE.
2. While we tested many different parameter values for both of PSO and DE, and selected the best results for both of them, GA gives the minimum set of features that yielded the highest accuracy among the three methods.

The results of PSO and DE are provided below in Tables S3.1 and S3.2, respectively, while the results of GA are provided in Table 2 in the main paper.

**Table S3.1.** The performance of the k-means clustering of 14 target AMP families using features selected by the Particle Swarm Optimization (PSO) algorithm.

| Target AMP family | Number of features | Accuracy | Sensitivity | Specificity | Precision | Jaccard Index | F-Measure | Entropy | Purity |
| --- | --- | --- | --- | --- | --- | --- | --- | --- | --- |
| Alpha-defensin | 101 | 99.34% | 91.18% | 99.72% | 93.94% | 86.11% | 92.54% | 0.0442 | 99.34% |
| Bacteriocin | 60 | 99.73% | 91.67% | 100.00% | 100.00% | 91.67% | 95.65% | 0.0226 | 99.73% |
| Beta-defensin | 92 | 98.80% | 85.37% | 99.58% | 92.11% | 79.55% | 88.61% | 0.0676 | 98.80% |
| Bombinin | 134 | 99.61% | 95.12% | 99.86% | 97.50% | 92.86% | 96.30% | 0.0271 | 99.61% |
| Cathelicidin | 204 | 97.21% | 59.26% | 98.62% | 61.54% | 43.24% | 60.38% | 0.1318 | 97.21% |
| Cecropin | 143 | 100.00% | 100.00% | 100.00% | 100.00% | 100.00% | 100.00% | 0 | 100.00% |
| Cyclotide (Bracelet subfamily) | 113 | 100.00% | 100.00% | 100.00% | 100.00% | 100.00% | 100.00% | 0 | 100.00% |
| DEFL | 90 | 98.80% | 89.19% | 99.30% | 86.84% | 78.57% | 88.00% | 0.0596 | 98.80% |
| FSAP (Brevinin subfamily) | 485 | 88.56% | 53.15% | 96.65% | 78.35% | 46.34% | 63.33% | 0.3161 | 91.81% |
| FSAP (Caerin subfamily) | 876 | 98.94% | 81.82% | 99.19% | 60.00% | 52.94% | 69.23% | 0.036 | 98.94% |
| FSAP (Dermaseptin subfamily) | 121 | 99.07% | 90.00% | 99.45% | 87.10% | 79.41% | 88.52% | 0.0498 | 99.07% |
| Invertebrate defensin (Type 1 subfamily) | 168 | 100.00% | 100.00% | 100.00% | 100.00% | 100.00% | 100.00% | 0 | 100.00% |
| Invertebrate defensin (Type 2 subfamily) | 209 | 98.94% | 61.54% | 99.59% | 72.73% | 50.00% | 66.67% | 0.0548 | 98.94% |
| Type A lantibiotic | 74 | 98.94% | 90.91% | 99.06% | 58.82% | 55.56% | 71.43% | 0.0299 | 98.94% |
| **Average** |  | 98.42% | 84.94% | 99.36% | 84.92% | 75.45% | 84.33% | 6.00% | 98.66% |

**Table S3.2.** The performance of the k-means clustering of 14 target AMP families using features selected by the Differential Evolution (DE) optimization algorithm.

| Target AMP family | Number of features | Accuracy | Sensitivity | Specificity | Precision | Jaccard Index | F-Measure | Entropy | Purity |
| --- | --- | --- | --- | --- | --- | --- | --- | --- | --- |
| Alpha-defensin | 73 | 99.20% | 88.24% | 99.72% | 93.75% | 83.33% | 90.91% | 0.0474 | 99.20% |
| Bacteriocin | 57 | 99.20% | 91.67% | 99.45% | 84.62% | 78.57% | 88.00% | 0.0443 | 99.20% |
| Beta-defensin | 85 | 98.01% | 80.49% | 99.02% | 82.50% | 68.75% | 81.48% | 0.0991 | 98.01% |
| Bombinin | 246 | 99.87% | 96.77% | 100.00% | 100.00% | 96.77% | 98.36% | 0.0097 | 99.87% |
| Cathelicidin | 129 | 97.08% | 55.56% | 98.62% | 60.00% | 40.54% | 57.69% | 0.135 | 97.08% |
| Cecropin | 211 | 98.94% | 86.67% | 99.45% | 86.67% | 76.47% | 86.67% | 0.0581 | 98.94% |
| Cyclotide (Bracelet subfamily) | 126 | 100.00% | 100.00% | 100.00% | 100.00% | 100.00% | 100.00% | 0 | 100.00% |
| DEFL | 72 | 97.88% | 72.97% | 99.16% | 81.82% | 62.79% | 77.14% | 0.1123 | 97.88% |
| FSAP (Brevinin subfamily) | 299 | 93.63% | 72.73% | 98.40% | 91.23% | 67.97% | 80.93% | 0.26 | 94.54% |
| FSAP (Caerin subfamily) | 392 | 99.74% | 81.82% | 100.00% | 100.00% | 81.82% | 90.00% | 0.0165 | 99.74% |
| FSAP (Dermaseptin subfamily) | 73 | 97.61% | 86.67% | 98.06% | 65.00% | 59.09% | 74.29% | 0.0835 | 97.61% |
| Invertebrate defensin (Type 1 subfamily) | 126 | 99.07% | 95.24% | 99.18% | 76.92% | 74.07% | 85.11% | 0.0359 | 99.07% |
| Invertebrate defensin (Type 2 subfamily) | 133 | 99.07% | 61.54% | 99.73% | 80.00% | 53.33% | 69.57% | 0.0478 | 99.07% |
| Type A lantibiotic | 52 | 95.88% | 81.82% | 96.09% | 23.68% | 22.50% | 36.73% | 0.0612 | 98.54% |
| **Average** |  | 98.23% | 82.30% | 99.06% | 80.44% | 69.00% | 79.78% | 7.22% | 98.48% |

**S3.2 Comparison of k-means clustering with Affinity Propagation (AP) clustering Algorithm**

We compared the performance of clustering 14 target AMP families using Affinity Propagation (AP) and k-means algorithms. The clustering results in Table S3.3 for AP as compared to the clustering results of k-means algorithm in Table 2 in the main paper shows the k-means clustering provided higher accuracy than AP for most of the AMP families.

**Table S3.3.** The performance of the Affinity Propagation (AP) clustering of 14 target AMP families using features selected by the Genetic Algorithm.

| Target AMP family | Number of features | Accuracy | Sensitivity | Specificity | Precision | Jaccard Index | F-Measure | Entropy | Purity |
| --- | --- | --- | --- | --- | --- | --- | --- | --- | --- |
| Alpha-defensin | 68 | 98.67% | 79.41% | 99.58% | 90.00% | 72.97% | 84.38% | 0.0719 | 98.67% |
| Bacteriocin | 70 | 99.20% | 83.33% | 99.73% | 90.91% | 76.92% | 86.96% | 0.0583 | 99.20% |
| Beta-defensin | 62 | 96.81% | 85.37% | 97.47% | 66.04% | 59.32% | 74.47% | 0.1165 | 96.81% |
| Bombinin | 113 | 100.00% | 100.00% | 100.00% | 100.00% | 100.00% | 100.00% | 0 | 100.00% |
| Cathelicidin | 71 | 98.54% | 70.37% | 99.59% | 86.36% | 63.33% | 77.55% | 0.0891 | 98.54% |
| Cecropin | 190 | 98.94% | 93.33% | 99.17% | 82.35% | 77.78% | 87.50% | 0.0469 | 98.94% |
| Cyclotide (Bracelet subfamily) | 93 | 100.00% | 100.00% | 100.00% | 100.00% | 100.00% | 100.00% | 0 | 100.00% |
| DEFL | 51 | 97.34% | 67.57% | 98.88% | 75.76% | 55.56% | 71.43% | 0.1484 | 97.34% |
| FSAP (Brevinin subfamily) | 254 | 96.15% | 83.92% | 99.02% | 95.24% | 80.54% | 89.22% | 0.2278 | 96.15% |
| FSAP (Caerin subfamily) | 241 | 98.67% | 81.82% | 98.92% | 52.94% | 47.37% | 64.29% | 0.0423 | 98.67% |
| FSAP (Dermaseptin subfamily) | 95 | 98.54% | 83.33% | 99.17% | 80.65% | 69.44% | 81.97% | 0.0739 | 98.54% |
| Invertebrate defensin (Type 1 subfamily) | 71 | 99.60% | 90.48% | 99.86% | 95.00% | 86.36% | 92.68% | 0.0276 | 99.60% |
| Invertebrate defensin (Type 2 subfamily) | 78 | 99.47% | 69.23% | 100.00% | 100.00% | 69.23% | 81.82% | 0.0269 | 99.47% |
| Type A lantibiotic | 48 | 99.20% | 54.55% | 99.87% | 85.71% | 50.00% | 66.67% | 0.0452 | 99.20% |
| **Average** |  | 98.65% | 81.62% | 99.38% | 85.78% | 72.06% | 82.78% | 6.96% | 98.65% |

**S3.3 Comparison of clustering using different distance measures**

We performed k-means clustering of 14 target AMP families using three different distance measures (correlation, cosine and city block) as shown in Tables S3.4-6. The comparison of these results with the clustering results using Euclidean distance (Table 2 in the main paper) shows that the clustering results using these measures are comparable to those obtained by the Euclidean distance, but better results with minimum number of features were obtained with the Euclidean distance.

**Table S3.4.** The performance of the k-means clustering (using city block distance measure) of 14 target AMP families using features selected by the Genetic Algorithm.

| Target AMP family | Number of features | Accuracy | Sensitivity | Specificity | Precision | Jaccard Index | F-Measure | Entropy | Purity |
| --- | --- | --- | --- | --- | --- | --- | --- | --- | --- |
| Alpha-defensin | 13 | 99.73% | 94.12% | 100.00% | 100.00% | 94.12% | 96.97% | 0.0209 | 99.73% |
| Bacteriocin | 11 | 99.87% | 95.83% | 100.00% | 100.00% | 95.83% | 97.87% | 0.0102 | 99.87% |
| Beta-defensin | 28 | 99.60% | 92.68% | 100.00% | 100.00% | 92.68% | 96.20% | 0.0251 | 99.60% |
| Bombinin | 17 | 100.00% | 100.00% | 100.00% | 100.00% | 100.00% | 100.00% | 0 | 100.00% |
| Cathelicidin | 39 | 99.47% | 88.89% | 99.86% | 96.00% | 85.71% | 92.31% | 0.037 | 99.47% |
| Cecropin | 42 | 100.00% | 100.00% | 100.00% | 100.00% | 100.00% | 100.00% | 0 | 100.00% |
| Cyclotide (Bracelet subfamily) | 4 | 100.00% | 100.00% | 100.00% | 100.00% | 100.00% | 100.00% | 0 | 100.00% |
| DEFL | 17 | 99.34% | 86.49% | 100.00% | 100.00% | 86.49% | 92.75% | 0.0485 | 99.34% |
| FSAP (Brevinin subfamily) | 110 | 97.92% | 90.21% | 99.68% | 98.47% | 88.97% | 94.16% | 0.1268 | 97.92% |
| FSAP (Caerin subfamily) | 484 | 99.60% | 81.82% | 99.87% | 90.00% | 75.00% | 85.71% | 0.0228 | 99.60% |
| FSAP (Dermaseptin subfamily) | 29 | 99.07% | 90.00% | 99.45% | 87.10% | 79.41% | 88.52% | 0.0524 | 99.07% |
| Invertebrate defensin (Type 1 subfamily) | 9 | 100.00% | 100.00% | 100.00% | 100.00% | 100.00% | 100.00% | 0 | 100.00% |
| Invertebrate defensin (Type 2 subfamily) | 8 | 99.34% | 61.54% | 100.00% | 100.00% | 61.54% | 76.19% | 0.0455 | 99.34% |
| Type A lantibiotic | 16 | 99.73% | 90.91% | 99.87% | 90.91% | 83.33% | 90.91% | 0.0156 | 99.73% |
| **Average** |  | 99.55% | 90.89% | 99.91% | 97.32% | 88.79% | 93.69% | 2.89% | 99.55% |

**Table S3.5.** The performance of the k-means clustering (using cosine distance measure) of 14 target AMP families using features selected by the Genetic Algorithm.

| Target AMP family | Number of features | Accuracy | Sensitivity | Specificity | Precision | Jaccard Index | F-Measure | Entropy | Purity |
| --- | --- | --- | --- | --- | --- | --- | --- | --- | --- |
| Alpha-defensin | 23 | 99.73% | 94.12% | 100.00% | 100.00% | 94.12% | 96.97% | 0.0187 | 99.73% |
| Bacteriocin | 23 | 99.87% | 95.83% | 100.00% | 100.00% | 95.83% | 97.87% | 0.0096 | 99.87% |
| Beta-defensin | 43 | 98.94% | 85.37% | 99.72% | 94.59% | 81.40% | 89.74% | 0.062 | 98.94% |
| Bombinin | 33 | 100.00% | 100.00% | 100.00% | 100.00% | 100.00% | 100.00% | 0 | 100.00% |
| Cathelicidin | 51 | 98.27% | 77.78% | 99.04% | 75.00% | 61.76% | 76.36% | 0.0899 | 98.27% |
| Cecropin | 55 | 100.00% | 100.00% | 100.00% | 100.00% | 100.00% | 100.00% | 0 | 100.00% |
| Cyclotide (Bracelet subfamily) | 17 | 100.00% | 100.00% | 100.00% | 100.00% | 100.00% | 100.00% | 0 | 100.00% |
| DEFL | 29 | 99.47% | 89.19% | 100.00% | 100.00% | 89.19% | 94.29% | 0.038 | 99.47% |
| FSAP (Brevinin subfamily) | 261 | 92.43% | 70.63% | 97.54% | 87.07% | 63.92% | 77.99% | 0.3356 | 92.43% |
| FSAP (Caerin subfamily) | 199 | 99.60% | 81.82% | 99.87% | 90.00% | 75.00% | 85.71% | 0.022 | 99.60% |
| FSAP (Dermaseptin subfamily) | 36 | 99.20% | 96.67% | 99.31% | 85.29% | 82.86% | 90.62% | 0.0367 | 99.20% |
| Invertebrate defensin (Type 1 subfamily) | 17 | 100.00% | 100.00% | 100.00% | 100.00% | 100.00% | 100.00% | 0 | 100.00% |
| Invertebrate defensin (Type 2 subfamily) | 44 | 100.00% | 100.00% | 100.00% | 100.00% | 100.00% | 100.00% | 0 | 100.00% |
| Type A lantibiotic | 37 | 99.60% | 100.00% | 99.60% | 78.57% | 78.57% | 88.00% | 0.0139 | 99.60% |
| **Average** |  | 99.08% | 92.24% | 99.65% | 93.61% | 87.33% | 92.68% | 4.47% | 99.08% |

**Table S3.6.** The performance of the k-means clustering (using correlation distance measure) of 14 target AMP families using features selected by the Genetic Algorithm.

| Target AMP family | Number of features | Accuracy | Sensitivity | Specificity | Precision | Jaccard Index | F-Measure | Entropy | Purity |
| --- | --- | --- | --- | --- | --- | --- | --- | --- | --- |
| Alpha-defensin | 24 | 99.73% | 94.12% | 100.00% | 100.00% | 94.12% | 96.97% | 0.0195 | 99.73% |
| Bacteriocin | 26 | 99.87% | 95.83% | 100.00% | 100.00% | 95.83% | 97.87% | 0.0095 | 99.87% |
| Beta-defensin | 41 | 99.60% | 92.68% | 100.00% | 100.00% | 92.68% | 96.20% | 0.0285 | 99.60% |
| Bombinin | 45 | 100.00% | 100.00% | 100.00% | 100.00% | 100.00% | 100.00% | 0 | 100.00% |
| Cathelicidin | 45 | 98.67% | 88.89% | 99.04% | 77.42% | 70.59% | 82.76% | 0.0603 | 98.67% |
| Cecropin | 36 | 100.00% | 100.00% | 100.00% | 100.00% | 100.00% | 100.00% | 0 | 100.00% |
| Cyclotide (Bracelet subfamily) | 30 | 100.00% | 100.00% | 100.00% | 100.00% | 100.00% | 100.00% | 0 | 100.00% |
| DEFL | 38 | 99.60% | 94.59% | 99.86% | 97.22% | 92.11% | 95.89% | 0.0291 | 99.60% |
| FSAP (Brevinin subfamily) | 370 | 88.84% | 45.45% | 99.02% | 91.55% | 43.62% | 60.75% | 0.3225 | 89.91% |
| FSAP (Caerin subfamily) | 112 | 99.87% | 90.91% | 100.00% | 100.00% | 90.91% | 95.24% | 0.0094 | 99.87% |
| FSAP (Dermaseptin subfamily) | 41 | 99.73% | 96.67% | 99.86% | 96.67% | 93.55% | 96.67% | 0.0183 | 99.73% |
| Invertebrate defensin (Type 1 subfamily) | 30 | 100.00% | 100.00% | 100.00% | 100.00% | 100.00% | 100.00% | 0 | 100.00% |
| Invertebrate defensin (Type 2 subfamily) | 35 | 99.60% | 92.31% | 99.73% | 85.71% | 80.00% | 88.89% | 0.0205 | 99.60% |
| Type A lantibiotic | 35 | 99.34% | 90.91% | 99.46% | 71.43% | 66.67% | 80.00% | 0.0255 | 99.34% |
| **Average** |  | 98.92% | 91.60% | 99.78% | 94.29% | 87.15% | 92.23% | 3.88% | 98.99% |

**S4. Supplementary Figures**


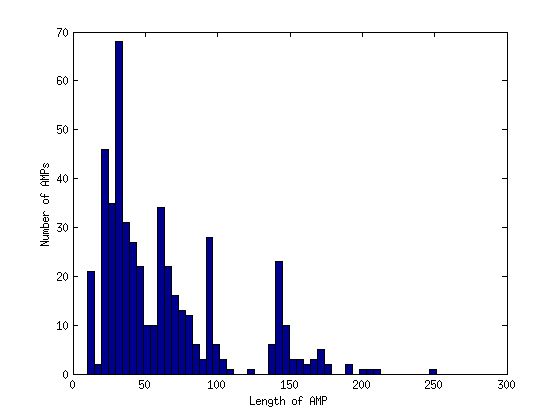


**Figure S1**. A histogram of the distribution of lengths of 478 AMPs from 14 target AMP families.


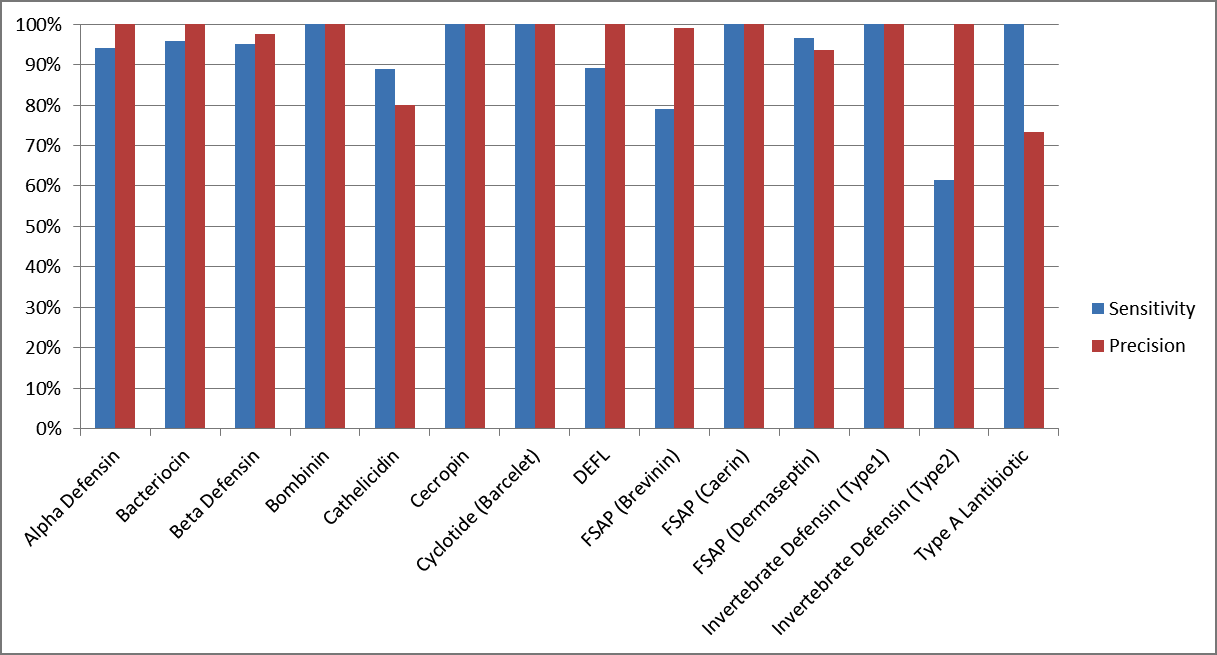


**Figure S2.**  Plot of sensitivity vs. specificity obtained from k-means clustering of 14 target AMP families using optimized set of selected features.


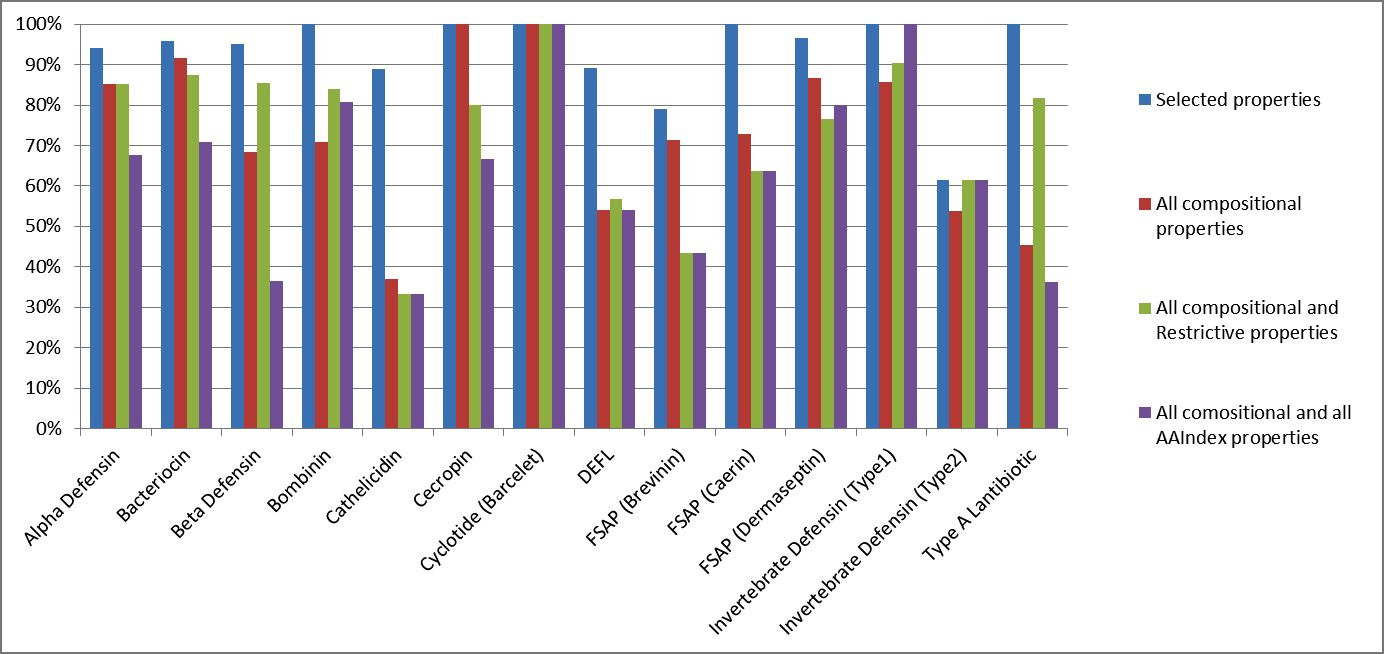


**Figure S3**. Bar plots of the sensitivity obtained from four different representations of AMPs.

**S5. Supplementary Text**

**Bacteriocins.** We found an enrichment of glycine, valine, asparagine and tyrosine in the n2, n2, n3 and n3 regions, respectively. Alanine (A) was found depleted in the N region. If we only consider the amino acids identified in our study as enriched, we pinpoint valine, glycine and tyrosine as key hydrophobic residues for bacteriocins. Findings reported by Jabrane et al. and Sit et al. partially support our results as they demonstrated that leucine and asparagine are enriched in bacteriocin serracin P ([Jabrane, et al., 2002](#_ENREF_7)). Oppegard et al. additionally demonstrated by mutation analysis that substituting tyrosine with glycine or leucine significantly decreases bactericidal activity ([Oppegard, et al., 2008](#_ENREF_12)).

**Bombinins.** We only identified a few properties related to composition and distance such as the enrichment of glycine and threonine (T) in the n1 and n4 regions, respectively, and of glycine and leucine in the C region. Our findings are in part supported by bombinins being characterized as glycine-rich and Zangger et al. demonstrated via NMR the presence of a glycine ridge that is believed to provide unique functionality ([Zangger, et al., 2008](#_ENREF_17)).

**Defensin-like (DEFL) peptides**. We found glutamic acid, serine, threonine, cysteine, cysteine, glutamic acid, cysteine, glycine and serine to be enriched in the n1, n1, n1, n2, n3, n3, n4, n4, n4 regions, respectively. Cysteine was enriched in the C region. We also found for the properties extracted from the n4 sub-region such as conformational parameter of inner helix ([Beghin and Dirkx, 1975](#_ENREF_1)) and ratio of average and computed composition ([Nakashima, et al., 1990](#_ENREF_11)) to be depleted. If we only consider the amino acids that our study identified as enriched we observe the N-terminal is negatively charged (glutamic acid) and enriched with cysteine and serine. Our findings are in part supported by Correa and Oguiura findings in which they produced a phylogenetic analysis of beta-defensin-like genes of Bothrops, Crotalus and Lachesis snakes and observed that these have conserved cysteine residues ([Correa and Oguiura, 2013](#_ENREF_5)).

**Frog skin active peptides.** We additionally discriminated sub-families, such as the brevinin, caerin and dermaseptin, belonging to the frog skin active peptide (FSAP) family. For the brevinin sub-family, we found enrichment of phenylalanine in the n1 region, and cysteine and isoleusine in the C region. Glutamic acid and serine were identified depleted in the n1 and n4 sub-regions, respectively. We also found the enrichment of physicochemical properties such as normalized positional residue frequency at helix termini N (n1 region), weights for alpha-helix at the window position of -6 (n2 region), normalized composition from fungi and plant (n2 region), normalized composition from mt-proteins (n3 region), pK(-COOH) (n3 region), normalized composition of membrane proteins (n4 region), weights for alpha-helix at the window position 1 (M region). Work by Pal *et al*. partially corroborates these findings as they demonstrated that replacement of the cysteine residues with serine in brevinin-1BYa, a cationic alpha-helical peptide present in skin secretions of the foothill yellow-legged frog Rana boylii, reduced its haemolytic activity and activities against Gram-negative bacteria and yeast species. However, high potency against Gram-positive bacteria was retained ([Pal, et al., 2006](#_ENREF_13)). Conlon *et al*. (2009) demonstrated via structure-activity relationship of the brevinin family peptides, that brevinin-1BLc is more potent than brevinin-1Ya and -1Yc and that the appreciably lower antimicrobial potencies of brevinin-1Ya and -1Yc correlates with decreased cationicity produced by the amino acid substitutions Lys(11)-->Asn (brevinin-1Ya) and Pro(14)-->Glu (brevinin-1Yc) ([Conlon, et al., 2009](#_ENREF_4)).

For the Caerin sub-family we identified fewer properties related to composition and distance of amino acid residues, such as the enrichment of valine in the C region, and enrichment of physicochemical properties such as normalized hydrophobicity scales for alpha-proteins (n1 region), weights for alpha-helix at the window position of 5 (n1 region), van der Waals parameter R0 (n1 region), surrounding hydrophobicity in turn (n1 region), Optical rotation (C region) and Normalized positional residue frequency at helix termini N" (C region). Our findings are supported in part as valine ([Wong, et al., 1997](#_ENREF_16)) was shown to play a role in the activity of caerins.

Lastly, dermaseptin sub-family showed enrichment of tryptophan in n1 and n4 regions. Alanine is enriched in a negatively charged M region, as well as glutamine and leucine in the C region. Properties that were depleted in this family include phenylalanine in the n1 region. We found enrichment of physicochemical properties such as weights for alpha-helix at the window position of -6 (n4 region), average interactions per side chain atom (M region) and AA composition of EXT of multi-spanning proteins (C region). Our finds are partially corroborated by Moll *et al*. demonstrating that tryptophan is important for this peptides ability to penetrate membranes ([Moll, et al., 2000](#_ENREF_10)). Lequin *et al*. further showed via comparison of dermaseptin B2 and S9 structures its common cationic amino acids to be lysine and glutamic acid, and key hydrophobic residues to be isoleucine, leucine and valine ([Lequin, et al., 2006](#_ENREF_8)). Moreover, Cao *et al*. compared the antimicrobial activities of recombinant adenoregulin with C-amidated terminus to that without an amidated C-terminus and demonstrated that the amide of glutamine at C-terminus increased its potency against microorganisms such as Tritirachium album and Saccharomyces cerevisiae ([Cao, et al., 2005](#_ENREF_2)).

**Type A lantibiotics.** We found valine and threonine enriched in the n2 and n4 regions, respectively, methionine in the M region and asparagine, cysteine, phenylalanine, serine and threonine enriched in the C region. Cysteine and methionine are also enriched in the entire sequence. Properties that appear depleted in Type A lantibiotics include alanine the N region and arginine in the entire sequence as well as the frequency of (>1 aa and <=6 aa) Distances of basic amino acids (RHK) in N region. Work by Slootweg *et al*. demonstrated that C-terminal modification of nisin does not deteriorate biological activity in sharp contrast to N-terminal modification ([Slootweg, et al., 2013](#_ENREF_14)). Since lantibiotics are a class of more extensively modified bacteriocins, characterized by the presence of lanthionine (Lan) and methyllanthionine (MeLan) ‘hinge’ regions that originate from cysteine and serine ([Dischinger, et al., 2014](#_ENREF_6); [Lohans and Vederas, 2014](#_ENREF_9)), our findings of enriched serine and threonine in this family compared to others are partially supported. Moreover, Chen *et al.* demonstrated using mutacin II that these hinge regions are essential for biological activity and biosynthesis or export of the peptide ([Chen, et al., 1998](#_ENREF_3)).

**Invertebrate defensins**. We additionally discriminated sub-familes (Type 1 and Type 2) of invertebrate defensins. For the invertebrate defensin Type 1 sub-family we found enrichment of aspartic acid, alanine, cysteine, cysteine, leucine, cysteine and valine in the n1, n2, n2, n4, n4, C and C regions, respectively. For the invertebrate defensin Type 2 sub-family (arthropods) we found histidine, cysteine, cysteine and threonine enriched in the n1, n2, n3 and C regions, respectively.

**References**

Beghin, F. and Dirkx, J. (1975) [Proceedings: A simple statistical method to predict protein conformations], *Archives internationales de physiologie et de biochimie*, **83**, 167-168.

Cao, W.*, et al.* (2005) Expression and purification of antimicrobial peptide adenoregulin with C-amidated terminus in Escherichia coli, *Protein expression and purification*, **40**, 404-410.

Chen, P.*, et al.* (1998) Structure-activity study of the lantibiotic mutacin II from Streptococcus mutans T8 by a gene replacement strategy, *Applied and environmental microbiology*, **64**, 2335-2340.

Conlon, J.M.*, et al.* (2009) Peptides with potent cytolytic activity from the skin secretions of the North American leopard frogs, Lithobates blairi and Lithobates yavapaiensis, *Toxicon : official journal of the International Society on Toxinology*, **53**, 699-705.

Correa, P.G. and Oguiura, N. (2013) Phylogenetic analysis of beta-defensin-like genes of Bothrops, Crotalus and Lachesis snakes, *Toxicon : official journal of the International Society on Toxinology*, **69**, 65-74.

Dischinger, J., Basi Chipalu, S. and Bierbaum, G. (2014) Lantibiotics: promising candidates for future applications in health care, *International journal of medical microbiology : IJMM*, **304**, 51-62.

Jabrane, A.*, et al.* (2002) Characterization of serracin P, a phage-tail-like bacteriocin, and its activity against Erwinia amylovora, the fire blight pathogen, *Applied and environmental microbiology*, **68**, 5704-5710.

Lequin, O.*, et al.* (2006) Dermaseptin S9, an alpha-helical antimicrobial peptide with a hydrophobic core and cationic termini, *Biochemistry*, **45**, 468-480.

Lohans, C.T. and Vederas, J.C. (2014) Structural characterization of thioether-bridged bacteriocins, *The Journal of antibiotics*, **67**, 23-30.

Moll, G.N.*, et al.* (2000) Comparison of the membrane interaction and permeabilization by the designed peptide Ac-MB21-NH2 and truncated dermaseptin S3, *Biochemistry*, **39**, 11907-11912.

Nakashima, H., Nishikawa, K. and Ooi, T. (1990) Distinct character in hydrophobicity of amino acid compositions of mitochondrial proteins, *Proteins*, **8**, 173-178.

Oppegard, C.*, et al.* (2008) Mutational analysis of putative helix-helix interacting GxxxG-motifs and tryptophan residues in the two-peptide bacteriocin lactococcin G, *Biochemistry*, **47**, 5242-5249.

Pal, T.*, et al.* (2006) Brevinin-1BYa: a naturally occurring peptide from frog skin with broad-spectrum antibacterial and antifungal properties, *International journal of antimicrobial agents*, **27**, 525-529.

Slootweg, J.C.*, et al.* (2013) Synthesis, antimicrobial activity, and membrane permeabilizing properties of C-terminally modified nisin conjugates accessed by CuAAC, *Bioconjugate chemistry*, **24**, 2058-2066.

Tan, P.-N., Steinbach, M. and Kumar, V. (2006) *Introduction to data mining*. Pearson Addison Wesley, Boston.

Wong, H., Bowie, J.H. and Carver, J.A. (1997) The solution structure and activity of caerin 1.1, an antimicrobial peptide from the Australian green tree frog, Litoria splendida, *European journal of biochemistry / FEBS*, **247**, 545-557.

Zangger, K.*, et al.* (2008) Structures of the glycine-rich diastereomeric peptides bombinin H2 and H4, *Toxicon : official journal of the International Society on Toxinology*, **52**, 246-254.

**S6. Selected Properties**

The following table shows the entire set of the selected compositional and physicochemical properties by the model. Different properties were selected for each AMP family.

| **AMP Family Name** | **Number of Selected Properties** | **Selected Properties** |
| --- | --- | --- |
| Alpha-defensin | 14 | **Basic Properties Related To Composition and Distance Frequency:**  Frequency of Amino Acid (Q) in n2 region  Frequency of Amino Acid (G) in n2 region  Frequency of Amino Acid (F) in n2 region  Frequency of Amino Acid (C) in n3 region  Frequency of Amino Acid (F) in n3 region  Frequency of Amino Acid (Y) in n3 region  Frequency of Amino Acid (N) in n4 region  Frequency of Amino Acid (C) in n4 region  Frequency of pairs Amino Acid (R) in M region  Frequency of Amino Acid (R) in C region  Frequency of Amino Acid (K) in C region  Frequency of Amino Acid (Y) in C region  **Properties Extracted from n1 Sub-Region:**  Short and medium range non-bonded energy per atom (Oobatake-Ooi, 1977)  **Properties Extracted from C Region:**  Weights for beta-sheet at the window position of 2 (Qian-Sejnowski, 1988) |
| Bacteriocin | 9 | **Basic Properties Related To Composition and Distance Frequency:**  Frequency of Amino Acid (G) in n2 region  Frequency of Amino Acid (V) in n2 region  Frequency of Amino Acid (A) in n3 region  Frequency of Amino Acid (N) in n3 region  Frequency of Amino Acid (Y) in n3 region  Frequency of (>16 aa and <=21 aa) Distances of basic amino acids (RHK) in N region  Frequency of Amino Acid (C) in the entire sequence region  **Properties Extracted from n4 Sub-Region:**  The number of atoms in the side chain labelled 2+1 (Charton-Charton, 1983)  **Properties Extracted from M Region:**  van der Waals parameter epsilon (Levitt, 1976) |
| Beta-defensin | 36 | **Basic Properties Related To Composition and Distance Frequency:**  Frequency of Amino Acid (R) in n1 region  Frequency of Amino Acid (H) in n1 region  Frequency of Amino Acid (N) in n2 region  Frequency of Amino Acid (I) in n2 region  Frequency of Amino Acid (L) in n2 region  Frequency of Amino Acid (V) in n2 region  Frequency of Amino Acid (L) in n3 region  Frequency of Amino Acid (M) in n3 region  Frequency of Amino Acid (F) in n3 region  Frequency of Amino Acid (P) in n3 region  Frequency of Amino Acid (T) in n3 region  Frequency of Amino Acid (W) in n3 region  Frequency of Amino Acid (Y) in n3 region  Frequency of Amino Acid (N) in n4 region  Frequency of Amino Acid (C) in n4 region  Frequency of Amino Acid (G) in n4 region  Frequency of Amino Acid (H) in n4 region  Frequency of Amino Acid (L) in n4 region  Frequency of Amino Acid (M) in n4 region  Frequency of Amino Acid (T) in n4 region  Frequency of Amino Acid (C) in M region  Frequency of Amino Acid (R) in C region  Frequency of Amino Acid (C) in C region  Frequency of Amino Acid (M) in C region  Frequency of Amino Acid (W) in C region  Frequency of Amino Acid (Y) in C region  Frequency of (1 aa) Distances of basic amino acids (RHK) in N region  Frequency of (>11 aa and <=16 aa) Distances of other non-hydrophobic amino acids (DNEQYSTC) in M region  Frequency of Amino Acid (W) in the entire sequence region  **Properties Extracted from n1 Sub-Region:**  The number of atoms in the side chain labelled 2+1 (Charton-Charton, 1983)  **Properties Extracted from n2 Sub-Region:**  A parameter of charge transfer donor capability (Charton-Charton, 1983)  **Properties Extracted from n3 Sub-Region:**  Average relative fractional occurrence in AL(i-1) (Rackovsky-Scheraga, 1982)  Normalized positional residue frequency at helix termini N" (Aurora-Rose,  **Properties Extracted from n4 Sub-Region:**  Average relative fractional occurrence in AL(i-1) (Rackovsky-Scheraga, 1982)  A parameter of charge transfer donor capability (Charton-Charton, 1983)  Linker propensity from all dataset (George-Heringa, 2003) |
| Bombinin | 13 | **Basic Properties Related To Composition and Distance Frequency:**  Frequency of Amino Acid (G) in n1 region  Frequency of Amino Acid (T) in n4 region  Frequency of Amino Acid (G) in C region  Frequency of Amino Acid (L) in C region  **Properties Extracted from n1 Sub-Region:**  Normalized frequency of beta-sheet (Crawford et al., 1973)  Weights for alpha-helix at the window position of -4 (Qian-Sejnowski, 1988)  **Properties Extracted from n4 Sub-Region:**  AA composition of membrane proteins (Nakashima et al., 1990)  Slope in regression analysis x 1.0E1 (Prabhakaran-Ponnuswamy, 1982)  Distribution of amino acid residues in the alpha-helices in thermophilic  Averaged turn propensities in a transmembrane helix (Monne et al., 1999)  **Properties Extracted from C Region:**  Normalized relative frequency of extended structure (Isogai et al., 1980)  Surface composition of amino acids in extracellular proteins of mesophiles  Weights for alpha-helix at the window position of -4 (Qian-Sejnowski, 1988) |
| Cathelicidin | 36 | **Basic Properties Related To Composition and Distance Frequency:**  Frequency of Amino Acid (R) in n1 region  Frequency of Amino Acid (H) in n1 region  Frequency of Amino Acid (Y) in n1 region  Frequency of Amino Acid (R) in n2 region  Frequency of Amino Acid (Q) in n2 region  Frequency of Amino Acid (T) in n2 region  Frequency of Amino Acid (Y) in n2 region  Frequency of Amino Acid (C) in n3 region  Frequency of Amino Acid (H) in n3 region  Frequency of Amino Acid (Y) in n3 region  Frequency of Amino Acid (C) in n4 region  Frequency of pairs Amino Acid (A) in M region  Frequency of Amino Acid (A) in C region  Frequency of Amino Acid (K) in C region  Frequency of Amino Acid (F) in C region  Frequency of Amino Acid (P) in C region  Frequency of Amino Acid (Y) in C region  **Properties Extracted from n3 Sub-Region:**  Atom-based hydrophobic moment (Eisenberg-McLachlan, 1986)  N.m.r. chemical shift of alpha-carbon (Fauchere et al., 1988)  Relative mutability (Jones et al., 1992)  Normalized frequency of beta-sheet, with weights (Levitt, 1978)  Frequency of occurrence in beta-bends (Lewis et al., 1971)  Normalized frequency of alpha region (Maxfield-Scheraga, 1976)  AA composition of membrane proteins (Nakashima et al., 1990)  Transmembrane regions of non-mt-proteins (Nakashima et al., 1990)  Weights for coil at the window position of -5 (Qian-Sejnowski, 1988)  Weights for coil at the window position of 5 (Qian-Sejnowski, 1988)  Relative preference value at N2 (Richardson-Richardson, 1988)  Principal property value z3 (Wold et al., 1987)  Normalized positional residue frequency at helix termini N4'(Aurora-Rose,  Normalized positional residue frequency at helix termini N3 (Aurora-Rose,  Alpha-helix propensity derived from designed sequences (Koehl-Levitt, 1999)  Linker propensity from 1-linker dataset (George-Heringa, 2003)  **Properties Extracted from C Region:**  Helix initiation parameter at posision i,i+1,i+2 (Finkelstein et al., 1991)  Helix termination parameter at posision j+1 (Finkelstein et al., 1991)  Relative preference value at C2 (Richardson-Richardson, 1988) |
| Cecropin | 33 | **Basic Properties Related To Composition and Distance Frequency:**  Frequency of Amino Acid (A) in n1 region  Frequency of Amino Acid (D) in n1 region  Frequency of Amino Acid (D) in n2 region  Frequency of Amino Acid (K) in n3 region  Frequency of Amino Acid (E) in n4 region  Frequency of Amino Acid (A) in C region  Frequency of (>16 aa and <=21 aa) Distances of other non-hydrophobic amino acids (DNEQYSTC) in M region  Frequency of Amino Acid (Y) in the entire sequence region  **Properties Extracted from n1 Sub-Region:**  Positive charge (Fauchere et al., 1988)  Conformational parameter of beta-turn (Beghin-Dirkx, 1975)  Bitterness (Venanzi, 1984)  **Properties Extracted from n2 Sub-Region:**  Residue volume (Bigelow, 1967)  Normalized frequency of beta-sheet (Crawford et al., 1973)  Entropy of formation (Hutchens, 1970)  **Properties Extracted from n3 Sub-Region:**  Information measure for extended without H-bond (Robson-Suzuki, 1976)  Weights for alpha-helix at the window position of 6 (Qian-Sejnowski, 1988)  Weights for coil at the window position of 3 (Qian-Sejnowski, 1988)  Free energy in beta-strand conformation (Munoz-Serrano, 1994)  Distribution of amino acid residues in the 18 non-redundant families of  **Properties Extracted from n4 Sub-Region:**  Hydration number (Hopfinger, 1971), Cited by Charton-Charton (1982)  Normalized positional residue frequency at helix termini N2 (Aurora-Rose,  Relative preference value at N3 (Richardson-Richardson, 1988)  Hydrophobicity coefficient in RP-HPLC, C4 with 0.1%TFA/MeCN/H2O (Wilce et al.  AA composition of mt-proteins (Nakashima et al., 1990)  **Properties Extracted from M Region:**  Averaged turn propensities in a transmembrane helix (Monne et al., 1999)  Propensity of amino acids within pi-helices (Fodje-Al-Karadaghi, 2002)  Linker propensity from long dataset (linker length is greater than 14  Optimized beta-structure-coil equilibrium constant (Oobatake et al., 1985)  Weights for alpha-helix at the window position of 5 (Qian-Sejnowski, 1988)  Weights for coil at the window position of 5 (Qian-Sejnowski, 1988)  Relative preference value at N" (Richardson-Richardson, 1988)  **Properties Extracted from C Region:**  Hydration number (Hopfinger, 1971), Cited by Charton-Charton (1982)  van der Waals parameter epsilon (Levitt, 1976) |
| Cyclotide (Bracelet sub-family) | 7 | **Basic Properties Related To Composition and Distance Frequency:**  Frequency of Amino Acid (E) in n4 region  Frequency of Amino Acid (G) in C region  **Properties Extracted from n1 Sub-Region:**  Normalized positional residue frequency at helix termini Cc (Aurora-Rose,  **Properties Extracted from n2 Sub-Region:**  The number of atoms in the side chain labelled 2+1 (Charton-Charton, 1983)  **Properties Extracted from C Region:**  The Kerr-constant increments (Khanarian-Moore, 1980)  Normalized frequency of turn in alpha+beta class (Palau et al., 1981)  Hydrostatic pressure asymmetry index, PAI (Di Giulio, 2005) |
| DEFL | 26 | **Basic Properties Related To Composition and Distance Frequency:**  Frequency of Amino Acid (E) in n1 region  Frequency of Amino Acid (K) in n1 region  Frequency of Amino Acid (S) in n1 region  Frequency of Amino Acid (T) in n1 region  Frequency of Amino Acid (C) in n2 region  Frequency of Amino Acid (C) in n3 region  Frequency of Amino Acid (E) in n3 region  Frequency of Amino Acid (L) in n3 region  Frequency of Amino Acid (Y) in n3 region  Frequency of Amino Acid (V) in n3 region  Frequency of Amino Acid (A) in n4 region  Frequency of Amino Acid (C) in n4 region  Frequency of Amino Acid (G) in n4 region  Frequency of Amino Acid (K) in n4 region  Frequency of Amino Acid (S) in n4 region  Frequency of pairs Amino Acid (C) in M region  Frequency of Amino Acid (C) in C region  Frequency of Amino Acid (W) in C region  Frequency of Amino Acid (V) in C region  Frequency of Amino Acid (K) in the entire sequence region  **Properties Extracted from n3 Sub-Region:**  The number of atoms in the side chain labelled 2+1 (Charton-Charton, 1983)  **Properties Extracted from n4 Sub-Region:**  A parameter of charge transfer donor capability (Charton-Charton, 1983)  Conformational parameter of inner helix (Beghin-Dirkx, 1975)  Residue accessible surface area in folded protein (Chothia, 1976)  Ratio of average and computed composition (Nakashima et al., 1990)  **Properties Extracted from M Region:**  The number of atoms in the side chain labelled 2+1 (Charton-Charton, 1983) |
| FSAP (Brevinin sub-family) | 118 | **Basic Properties Related To Composition and Distance Frequency:**  Frequency of Amino Acid (R) in n1 region  Frequency of Amino Acid (E) in n1 region  Frequency of Amino Acid (F) in n1 region  Frequency of Amino Acid (V) in n1 region  Frequency of Amino Acid (D) in n2 region  Frequency of Amino Acid (Q) in n2 region  Frequency of Amino Acid (P) in n2 region  Frequency of Amino Acid (W) in n2 region  Frequency of Amino Acid (R) in n3 region  Frequency of Amino Acid (C) in n3 region  Frequency of Amino Acid (S) in n4 region  Frequency of Amino Acid (W) in n4 region  Frequency of Amino Acid (V) in n4 region  Frequency of pairs Amino Acid (M) in M region  Frequency of Amino Acid (C) in C region  Frequency of Amino Acid (I) in C region  Frequency of Amino Acid (S) in C region  Frequency of Amino Acid (Y) in C region  **Properties Extracted from n1 Sub-Region:**  Normalized flexibility parameters (B-values) for each residue surrounded by  Weights for beta-sheet at the window position of 3 (Qian-Sejnowski, 1988)  Weights for coil at the window position of 3 (Qian-Sejnowski, 1988)  Average relative fractional occurrence in EL(i-1) (Rackovsky-Scheraga, 1982)  Normalized positional residue frequency at helix termini N4'(Aurora-Rose,  Average relative fractional occurrence in E0(i-1) (Rackovsky-Scheraga, 1982)  A parameter defined from the residuals obtained from the best correlation of  Free energies of transfer of AcWl-X-LL peptides from bilayer interface to  N.m.r. chemical shift of alpha-carbon (Fauchere et al., 1988)  Information measure for C-terminal turn (Robson-Suzuki, 1976)  Slopes tripeptide FDPB PARSE neutral (Avbelj, 2000)  Screening coefficients gamma, local (Avbelj, 2000)  **Properties Extracted from n2 Sub-Region:**  Weights for alpha-helix at the window position of -6 (Qian-Sejnowski, 1988)  Atom-based hydrophobic moment (Eisenberg-McLachlan, 1986)  Direction of hydrophobic moment (Eisenberg-McLachlan, 1986)  Side chain angle theta(AAR) (Levitt, 1976)  Localized electrical effect (Fauchere et al., 1988)  Normalized composition from fungi and plant (Nakashima et al., 1990)  Frequency of the 4th residue in turn (Chou-Fasman, 1978b)  Normalized positional residue frequency at helix termini N'(Aurora-Rose,  Normalized frequency of extended structure (Burgess et al., 1974)  **Properties Extracted from n3 Sub-Region:**  Helix termination parameter at posision j-2,j-1,j (Finkelstein et al., 1991)  Normalized composition of mt-proteins (Nakashima et al., 1990)  Residue accessible surface area in folded protein (Chothia, 1976)  The number of atoms in the side chain labelled 1+1 (Charton-Charton, 1983)  Normalized frequency of alpha region (Maxfield-Scheraga, 1976)  pK (-COOH) (Jones, 1975)  Normalized frequency of beta-sheet in alpha+beta class (Palau et al., 1981)  Information measure for extended without H-bond (Robson-Suzuki, 1976)  Weights for alpha-helix at the window position of 6 (Qian-Sejnowski, 1988)  Weights for coil at the window position of 4 (Qian-Sejnowski, 1988)  Optimized beta-structure-coil equilibrium constant (Oobatake et al., 1985)  Linker propensity from helical (annotated by DSSP) dataset (George-Heringa,  Optimized side chain interaction parameter (Oobatake et al., 1985)  Free energies of transfer of AcWl-X-LL peptides from bilayer interface to  Normalized frequency of turn (Crawford et al., 1973)  Information measure for C-terminal turn (Robson-Suzuki, 1976)  Normalized frequency of extended structure (Burgess et al., 1974)  **Properties Extracted from n4 Sub-Region:**  van der Waals parameter epsilon (Levitt, 1976)  Normalized composition of membrane proteins (Nakashima et al., 1990)  Electron-ion interaction potential (Veljkovic et al., 1985)  Relative preference value at C1 (Richardson-Richardson, 1988)  Relative population of conformational state E (Vasquez et al., 1983)  Frequency of the 3rd residue in turn (Chou-Fasman, 1978b)  Free energy in beta-strand conformation (Munoz-Serrano, 1994)  **Properties Extracted from M Region:**  Weights for beta-sheet at the window position of -3 (Qian-Sejnowski, 1988)  Bitterness (Venanzi, 1984)  Information measure for extended without H-bond (Robson-Suzuki, 1976)  Entropy of formation (Hutchens, 1970)  Relative preference value at C2 (Richardson-Richardson, 1988)  Normalized positional residue frequency at helix termini N'(Aurora-Rose,  Residue accessible surface area in folded protein (Chothia, 1976)  Normalized frequency of beta-sheet (Chou-Fasman, 1978b)  Relative mutability (Dayhoff et al., 1978b)  Localized electrical effect (Fauchere et al., 1988)  Weights for alpha-helix at the window position of 1 (Qian-Sejnowski, 1988)  Smoothed upsilon steric parameter (Fauchere et al., 1988)  The Kerr-constant increments (Khanarian-Moore, 1980)  Weights for alpha-helix at the window position of -4 (Qian-Sejnowski, 1988)  Average relative fractional occurrence in A0(i-1) (Rackovsky-Scheraga, 1982)  Relative preference value at C4 (Richardson-Richardson, 1988)  Normalized positional residue frequency at helix termini N" (Aurora-Rose,  Hydrophobicity coefficient in RP-HPLC, C18 with 0.1%TFA/MeCN/H2O (Wilce et  Information value for accessibility; average fraction 35% (Biou et al., 1988)  The Chou-Fasman parameter of the coil conformation (Charton-Charton, 1983)  Weights for alpha-helix at the window position of 6 (Qian-Sejnowski, 1988)  Weights for beta-sheet at the window position of 6 (Qian-Sejnowski, 1988)  Weights for coil at the window position of 4 (Qian-Sejnowski, 1988)  Average relative fractional occurrence in E0(i) (Rackovsky-Scheraga, 1982)  Free energies of transfer of AcWl-X-LL peptides from bilayer interface to  Weights for coil at the window position of -3 (Qian-Sejnowski, 1988)  Thermodynamic beta sheet propensity (Kim-Berg, 1993)  Linker propensity from long dataset (linker length is greater than 14  Optimized average non-bonded energy per atom (Oobatake et al., 1985)  Hydropathy scale based on self-information values in the two-state model (50%  **Properties Extracted from C Region:**  Weights for alpha-helix at the window position of -6 (Qian-Sejnowski, 1988)  Atom-based hydrophobic moment (Eisenberg-McLachlan, 1986)  Relative preference value at C3 (Richardson-Richardson, 1988)  Proportion of residues 95% buried (Chothia, 1976)  Weights for alpha-helix at the window position of -3 (Qian-Sejnowski, 1988)  Weights for coil at the window position of -4 (Qian-Sejnowski, 1988)  Average relative fractional occurrence in ER(i-1) (Rackovsky-Scheraga, 1982)  Slopes tripeptide FDPB PARSE neutral (Avbelj, 2000)  Hydrophobicity index (Argos et al., 1982)  Short and medium range non-bonded energy per atom (Oobatake-Ooi, 1977)  Value of theta(i-1) (Rackovsky-Scheraga, 1982)  Relative preference value at N3 (Richardson-Richardson, 1988)  Information measure for C-terminal turn (Robson-Suzuki, 1976)  Normalized positional residue frequency at helix termini C" (Aurora-Rose,  p-Values of thermophilic proteins based on the distributions of B values  Weights for alpha-helix at the window position of -4 (Qian-Sejnowski, 1988)  Weights for beta-sheet at the window position of 2 (Qian-Sejnowski, 1988)  Normalized positional residue frequency at helix termini C5 (Aurora-Rose,  Hydropathy scale based on self-information values in the two-state model (50%  Normalized frequency of N-terminal helix (Chou-Fasman, 1978b)  Relative population of conformational state A (Vasquez et al., 1983)  Normalized positional residue frequency at helix termini N2 (Aurora-Rose,  Normalized frequency of beta-sheet in alpha+beta class (Palau et al., 1981)  Hydrophobicity coefficient in RP-HPLC, C18 with 0.1%TFA/2-PrOH/MeCN/H2O  AA composition of EXT of multi-spanning proteins (Nakashima-Nishikawa, 1992) |
| FSAP (Caerin sub-family) | 28 | **Basic Properties Related To Composition and Distance Frequency:**  Frequency of Amino Acid (V) in C region  **Properties Extracted from n1 Sub-Region:**  Normalized hydrophobicity scales for alpha-proteins (Cid et al., 1992)  Weights for alpha-helix at the window position of 5 (Qian-Sejnowski, 1988)  van der Waals parameter R0 (Levitt, 1976)  Surrounding hydrophobicity in turn (Ponnuswamy et al., 1980)  Free energies of transfer of AcWl-X-LL peptides from bilayer interface to  **Properties Extracted from n2 Sub-Region:**  The Chou-Fasman parameter of the coil conformation (Charton-Charton, 1983)  Relative preference value at C5 (Richardson-Richardson, 1988)  Normalized frequency of isolated helix (Tanaka-Scheraga, 1977)  **Properties Extracted from n3 Sub-Region:**  Information measure for N-terminal turn (Robson-Suzuki, 1976)  Electron-ion interaction potential (Veljkovic et al., 1985)  **Properties Extracted from n4 Sub-Region:**  Transmembrane regions of non-mt-proteins (Nakashima et al., 1990)  Weights for alpha-helix at the window position of -6 (Qian-Sejnowski, 1988)  Average relative fractional occurrence in EL(i-1) (Rackovsky-Scheraga, 1982)  Relative preference value at C" (Richardson-Richardson, 1988)  **Properties Extracted from M Region:**  Helix initiation parameter at posision i-1 (Finkelstein et al., 1991)  Normalized relative frequency of helix end (Isogai et al., 1980)  Weights for beta-sheet at the window position of -5 (Qian-Sejnowski, 1988)  Relative preference value at C2 (Richardson-Richardson, 1988)  Slopes dekapeptide, FDPB VFF neutral (Avbelj, 2000)  Weights for coil at the window position of -6 (Qian-Sejnowski, 1988)  Weights for coil at the window position of 6 (Qian-Sejnowski, 1988)  Information measure for N-terminal turn (Robson-Suzuki, 1976)  **Properties Extracted from C Region:**  Optical rotation (Fasman, 1976)  Beta-strand indices (Geisow-Roberts, 1980)  Composition (Grantham, 1974)  Normalized frequency of beta-sheet in alpha+beta class (Palau et al., 1981)  Normalized positional residue frequency at helix termini N" (Aurora-Rose, |
| FSAP (Dermaseptin sub-family) | 25 | **Basic Properties Related To Composition and Distance Frequency:**  Frequency of Amino Acid (D) in n1 region  Frequency of Amino Acid (F) in n1 region  Frequency of Amino Acid (W) in n1 region  Frequency of Amino Acid (D) in n2 region  Frequency of Amino Acid (V) in n2 region  Frequency of Amino Acid (D) in n3 region  Frequency of Amino Acid (M) in n3 region  Frequency of Amino Acid (R) in n4 region  Frequency of Amino Acid (W) in n4 region  Frequency of Amino Acid (V) in M region  Frequency of pairs Amino Acid (A) in M region  Frequency of Amino Acid (C) in C region  Frequency of Amino Acid (Q) in C region  Frequency of Amino Acid (L) in C region  Frequency of Amino Acid (K) in C region  Frequency of Amino Acid (M) in C region  **Properties Extracted from n1 Sub-Region:**  Optimized average non-bonded energy per atom (Oobatake et al., 1985)  Bitterness (Venanzi, 1984)  **Properties Extracted from n2 Sub-Region:**  Helix termination parameter at posision j-2,j-1,j (Finkelstein et al., 1991)  **Properties Extracted from n4 Sub-Region:**  Weights for alpha-helix at the window position of -6 (Qian-Sejnowski, 1988)  **Properties Extracted from M Region:**  van der Waals parameter epsilon (Levitt, 1976)  Average interactions per side chain atom (Warme-Morgan, 1978)  **Properties Extracted from C Region:**  Entropy of formation (Hutchens, 1970)  AA composition of EXT of multi-spanning proteins (Nakashima-Nishikawa, 1992)  Information measure for extended without H-bond (Robson-Suzuki, 1976) |
| Invertebrate defensin (Type 1 sub-family) | 14 | **Basic Properties Related To Composition and Distance Frequency:**  Frequency of Amino Acid (D) in n1 region  Frequency of Amino Acid (A) in n2 region  Frequency of Amino Acid (C) in n2 region  Frequency of Amino Acid (C) in n4 region  Frequency of Amino Acid (L) in n4 region  Frequency of Amino Acid (C) in C region  Frequency of Amino Acid (S) in C region  Frequency of Amino Acid (V) in C region  **Properties Extracted from n4 Sub-Region:**  Linker propensity from 3-linker dataset (George-Heringa, 2003)  Conformational parameter of inner helix (Beghin-Dirkx, 1975)  The Chou-Fasman parameter of the coil conformation (Charton-Charton, 1983)  Frequency of occurrence in beta-bends (Lewis et al., 1971)  Retention coefficient in HPLC, pH2.1 (Meek, 1980)  Principal component III (Sneath, 1966) |
| Invertebrate defensin (Type 2 sub-family) | 9 | **Basic Properties Related To Composition and Distance Frequency:**  Frequency of Amino Acid (H) in n1 region  Frequency of Amino Acid (C) in n2 region  Frequency of Amino Acid (C) in n3 region  Frequency of Amino Acid (T) in C region  **Properties Extracted from n1 Sub-Region:**  AA composition of EXT of multi-spanning proteins (Nakashima-Nishikawa, 1992)  Value of theta(i) (Rackovsky-Scheraga, 1982)  Loss of Side chain hydropathy by helix formation (Roseman, 1988)  Principal component IV (Sneath, 1966)  **Properties Extracted from n2 Sub-Region:**  Loss of Side chain hydropathy by helix formation (Roseman, 1988) |
| Type A lantibiotic | 26 | **Basic Properties Related To Composition and Distance Frequency:**  Frequency of Amino Acid (N) in n1 region  Frequency of Amino Acid (D) in n1 region  Frequency of Amino Acid (C) in n1 region  Frequency of Amino Acid (S) in n1 region  Frequency of Amino Acid (A) in n2 region  Frequency of Amino Acid (C) in n2 region  Frequency of Amino Acid (G) in n2 region  Frequency of Amino Acid (V) in n2 region  Frequency of Amino Acid (N) in n3 region  Frequency of Amino Acid (T) in n4 region  Frequency of Amino Acid (M) in M region  Frequency of pairs Amino Acid (C) in M region  Frequency of pairs Amino Acid (M) in M region  Frequency of pairs Amino Acid (F) in M region  Frequency of Amino Acid (N) in C region  Frequency of Amino Acid (C) in C region  Frequency of Amino Acid (Q) in C region  Frequency of Amino Acid (K) in C region  Frequency of Amino Acid (F) in C region  Frequency of Amino Acid (S) in C region  Frequency of Amino Acid (T) in C region  Frequency of Amino Acid (Y) in C region  Frequency of (>1 aa and <=6 aa) Distances of basic amino acids (RHK) in N region  Frequency of Amino Acid (R) in the entire sequence region  Frequency of Amino Acid (C) in the entire sequence region  Frequency of Amino Acid (M) in the entire sequence region |
